# Protamine-Based Nanotherapeutics for Gene Delivery to Glioblastoma Cells

**DOI:** 10.1021/acs.molpharmaceut.4c01269

**Published:** 2025-04-02

**Authors:** Sheila Barrios-Esteban, Sonia Reimóndez-Troitiño, Pablo Cabezas-Sainz, María de la Fuente, Laura Sánchez, Ruman Rahman, Cameron Alexander, Marcos Garcia-Fuentes, Noemi S. Csaba

**Affiliations:** † Center for Research in Molecular Medicine and Chronic Diseases (CiMUS), 16780 University of Santiago de Compostela, Campus Vida, 15706 Santiago de Compostela, Spain; ‡ Department Pharmacology, Pharmacy and Pharmaceutical Technology, School of Pharmacy, University of Santiago de Compostela, Campus Vida, 15706 Santiago de Compostela, Spain; § School of Veterinary, University of Santiago de Compostela, Campus de Lugo, 27002 Lugo, Spain; ∥ Health Research Institute of Santiago de Compostela (IDIS), 15706 Santiago de Compostela, Spain; ⊥ Children’s Brain Tumor Research Centre (CBTR) and Biodiscovery Institute (BDI), University of Nottingham, University Park, NG7 2RD Nottingham, U.K.; # School of Pharmacy, ^b^Boots Science Building (BSB), University of Nottingham, East Dr, NG7 2TQ Nottingham, U.K.

**Keywords:** gene delivery, protamine nanoparticles, dextran sulfate, glioblastoma spheroids, zebrafish models

## Abstract

Isocitrate dehydrogenase wild-type glioblastoma is the most aggressive primary brain tumor classified as grade 4 of malignancy. Standard treatment, combining surgical resection, radiotherapy, and chemotherapy, often leads to severe side effects, with the emergence of tumor recurrence in all cases. Nucleic acid-based therapy has emerged as a promising strategy for cancer treatment. Non-viral nanosystems have become the vehicles of choice for gene delivery, due to their efficient nucleic acid encapsulation, protection, and intracellular transport. This work explores the potential of a formulation of low molecular weight protamine (LMWP) and dextran sulfate for gene delivery. The nanoparticles (NPs) were evaluated in terms of particle size, surface charge, morphology, and capacity to condense different nucleic acids. NPs formed by ionic complexation resulted in a homogeneous population of spherical particles with a low polydispersity index (PDI), small size, and positive surface charge. Competitive displacement assay demonstrated that the NPs could condense nucleic acids without alterations in their morphology and physicochemical characteristics, even after long-term storage. The efficacy of this formulation as a gene delivery system was evaluated *in vitro* in different glioblastoma cell lines and three-dimensional (3D) spheroids and *in vivo* using zebrafish models, showing negligible toxicity, efficient internalization, and consistent expression of fluorescent/luminescent proteins. Overall, these cationic polymeric NPs show promising features for their use as non-viral gene delivery vehicles for glioblastoma treatments.

## Graphical abstract

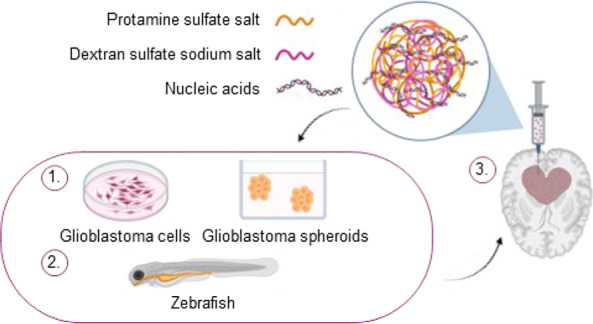

## 1 Introduction

Isocitrate dehydrogenase wild-type (WT) glioblastoma is the most common histologic subtype of gliomas classified as grade 4 of malignancy by the World Health Organization (WHO). The ineffectiveness of conventional treatment which comprises surgery, followed by concomitant radiotherapy and temozolomide chemotherapy, renders glioblastoma a hard-to-treat cancer and represents a critical global unmet clinical need.^1^

Nucleic acid-based therapy is considered a promising new option, but the delivery of exogenous genetic material requires overcoming numerous extra- and intracellular obstacles to reach the cellular cytoplasm or nucleus.^2,3^ To overcome these limitations, non-viral delivery systems offer an ability to protect the genetic material, promoting the intracellular delivery of genes. Cationic polymers are one of the most common materials studied in gene delivery due to their versatility, biodegradability, easy synthesis, and scalable production.^4^ Moreover, their capacity to interact with blood–brain-barrier (BBB) endothelial cell membranes facilitates their endocytosis.^5^ Cationic polymers can form polyplexes with negatively charged nucleic acids via electrostatic interactions. Among them, poly­(2-*N*-(dimethylaminoethyl)­methacrylate) (PDMAEMA), poly­(l-lysine) (PLL), and especially polyethylenimine (PEI) polyplexes are considered the “gold standard” for nucleic acid delivery due to their high transfection efficiency.^6,7^ However, their high cytotoxicity still limits their *in vivo* application.

Considering this and the variety of polymers available to formulate gene delivery nanosystems, the present work is focused on the natural and safe biomaterial protamine (Pr). Protamine is a cell-penetrating peptide (CPP) with low molecular weight (Mw = 5 kDa)^8^ and high capacity to condense different nucleic acids such as DNA, micro-RNAs (miRNAs), and small interfering RNAs (siRNAs).^9^ Protamine exhibits membrane translocation properties that are attributed to its arginine-rich sequence. These properties have been used for the intracellular administration of proteins and genes.^10,11^ Protamine is considered biologically safe and has been clinically approved by the Food and Drug Administration (FDA).^8^ The other material used for nanoparticle (NP) formulation is dextran sulfate (Dx), a negatively charged polysaccharide with variable molecular weight.^12^ Low molecular weight dextran sulfate (Mw = 5 kDa) is considered a promising material for the association to other polymers in controlled release systems, due to its capacity for ionotropic gelation, biocompatibility, and biodegradability.^13^ With this information, we hypothesize that the complexation of protamine and dextran may lead to a more effective gene delivery vector for the treatment of glioblastoma. Previous studies, particularly those by Thomas et al.,^14^ have shown that combining these two polymers creates a promising system for DNA delivery and the co-delivery of drugs or nucleic acids, such as docetaxel and siRNA.^15^ Moreover, a combination of Pr/Dx was also used as a coating on lipid NPs to enhance their transfection efficiency and mucopenetrating properties.^16–18^ The present work explores the potential application of this protamine-based nanosystem for glioblastoma treatment, as no previous research has specifically investigated this issue. Pr/Dx NPs, characterized by their safety and biocompatibility, have demonstrated remarkable ability to protect DNA from degradation by serum DNase I and lysosomal enzymes. Considering this, along with the ability to condense genetic material and the internalization capacity of protamine as a CPP, we consider this nanosystem to be a promising candidate for gene delivery. To validate these properties, our study stands out for the use of advanced preclinical tools, including patient-derived 3D glioblastoma models.

Important to our research was the incorporation of 3D glioblastoma spheroid models to achieve *in vivo*-like conditions for the evaluation of these NPs. The architecture of the spheroids is suitable for closely mimicking the tumor morphology under *in vitro* conditions. Indeed, the spheroids resemble solid tumors in many respects, such as structural organization, cell layer assembly, hypoxia, and nutrient gradients.^19,20^ Finally, to complement our *in vitro* findings, the toxicity of this formulation was also evaluated in advanced preclinical models using zebrafish embryos. This simple and reliable model is considered as an intermediate between *in vitro* and *in vivo* rodent models, where their transparency offers the possibility of studying the interaction of the NPs at the cellular level.^21^

## 2 Materials and Methods

### 2.1 Materials

Protamine sulfate salt (Mw-5 kDa, European Pharmacopeia (EP) grade) was obtained from Yuki Gosei Kogyo. Ltd. Dextran sulfate sodium salt from Leuconostoc spp., agarose, heparin sodium salt from porcine mucosa (25 KU), loading-buffer 10× (LB) Agar, Tris–Acetate–EDTA (TAE) Buffer 10×, sodium chloride (NaCl) BioXtra ≥99.5% (AT), sodium dodecyl sulfate (SDS), the Fluoromount aqueous mounting medium, 4% (v/v) paraformaldehyde, phosphotungstic acid (sodium salt), the Luciferase Reporter Gene Detection kit (LUC1), and kanamycin were purchased in Sigma-Aldrich. Triton-100X and SYBR Gold Nucleic Acid Gel Stain 50× were purchased from Scharlab S.L. Cellular membrane (Mw-3.5 kDa, 16 mm dry, I.D 35 feet, SnakeSkin), diethyl pyrocarbonate, ultrapure (DEPC) > 97%, LIVE/DEAD Fixable Aqua Dead Cell Stain Kit 405 nm excitation (200 assays), PrestoBlue Cell Viability reagent, Lipofectamine 2000 Transfection Reactive, and micro-BCA Protein Assay kit were from Thermo Fisher Scientific. The 5-carboxytetramethylrhodamine succinimidyl ester single isomer (5-TAMRA) and 4′,6-diamino-2-phenylindole (DAPI) were purchased from Emp-Biotech and Biochem, respectively. CellTiter Blue Cell Viability Assay was obtained from Promega. The decontamination solution RNase-free AWAY and UltraPure DNase/RNase-Free Distilled Water were obtained from Molecular Bioproduct. The 7-aminoactinomycin D (7-AAD) Viability Staining Solution was obtained from Invitrogen. The Luciferase Reporter Gene Assay High Sensitivity kit was purchased from Roche. 10% (v/v) neutral buffered formalin was obtained from Bio-Optica. Sodium bicarbonate (NaHCO_3_) 99% was purchased from Alfa Aesar, and dimethyl sulfoxide (DMSO) and the ethanol gradient grade for liquid chromatography (≥99%) were purchased from Merck Millipore. The μ-Slide-8-well (1.5 polymer coverslip, tissue culture-sterilized) was purchased from Ibidi.

Regarding the cell culture, Dulbecco’s modified Eagle’s medium 1× (DMEM) ([+] 4.5 g/L d-glucose and 1 g/L d-glucose, [+]­pyruvate, [+]l-glutamine), Dulbecco’s modified Eagle’s medium 1× (DMEM) ([+] 1 g/L d-glucose, [+]­pyruvate, [−]l-glutamine, no phenol red), opti-minimum essential medium I 1× reduced serum medium (Opti-MEM) ([+]­HEPES, [+]­2.4 g/L sodium bicarbonate, [+]l-glutamine), fetal bovine serum qualified (FBS), penicillin–streptomycin (P/S) ([+]­10,000 units/mL penicillin, [+]­10,000 μg/mL streptomycin), l-glutamine solution (200 mM, sterile-filtered, BioXtra), and 0.05% Trypsin 1×–EDTA were purchased from Gibco (Life Technologies). Dulbecco’s phosphate buffered salt solution 10× (DPBS) with calcium chloride and magnesium chloride ions, and modified Hanks balanced salt solution (HBSS) with phenol red and free of calcium and magnesium were also obtained from the latter supplier. The phosphate buffered salt solution 10× (PBS) and Luria–Bertani medium were prepared in the laboratory.

The model plasmid pEGFP-Luc was a kind donation by the Cell Cycle and Oncology group (CYCLON, University of Santiago de Compostela) and was produced and purified by a PureLink HiPure Expi Plasmid Gigaprep Kit from Invitrogen. The RNAs were purchased from Eurofins MWG Operon (Table 1).

**Table 1:** RNA Sequences Used

RNA	sequence
scrambled siRNA	5′ AGGUAGUGUAAUCGCCUUG 3′
	5′ CAAGGCGAUUACACUACCU 3′
Cy5-siRNA	5′ Cy5-AGGUAGUGUAAUCGCCUUG 3′
	5′ CAAGGCGAUUACACUACCU 3′
scrambled miRNA	5′ UUCUCCGAACGUUGUCACGUUU 3′
miRNA-145	5′ GUCCAGUUUUCCCAGGAAUCCCU 3′

MicroRNA Purification Kit was from Norgen Biotek Corporation. qScriptTM microRNA cDNA Synthesis kit, PerfeCta Universal PCR Primer and PerfeCta SYBRGreen SuperMix, and Low ROXTM were supplied for Quantabio, VWR International Eurolab. Primers, hsa-miR145-5p (5′GUCCAGUUUUCCCAGGAAUCCCU3′), and the small housekeeping RNA control primer RNU6 (5′CTCGCTTCGGCAGCACA3′, 5′AACGCTTCACGAATTTGCGT3′) were bought from IDT and Fisher Scientific, respectively.

### 2.2 Formulation of Protamine NPs

Pr/Dx NPs were prepared by an ionic cross-linking method previously described by our research group.^22^ Briefly, stock solutions of protamine and dextran were prepared in Milli-Q water at concentrations of 2 and 4 mg/mL, respectively. Based on previous results from the group, the NP prototype based on a 4:1 (w/w) Pr/Dx ratio was selected for this work.^22^ For the NP formation, a volume of 0.250 mL of dextran solution was added dropwise over 0.5 mL of a solution with 1 mg of protamine. This addition was performed under magnetic stirring at 500 rpm at room temperature (RT). After that, the formulation was incubated for 5 min at RT for complete formation of the NPs, indicated by the appearance of an opalescent suspension.

### 2.3 Morphological and Physicochemical Characterization

The NPs were characterized for mean particle size (hydrodynamic diameter), polydispersity index (PDI), derived count rate (DCR), and surface charge. The size, PDI, and DCR were measured by Photon Correlation Spectroscopy (PCS), and the zeta potential was measured by Laser Doppler Anemometry (LDA) at 25 °C using a detection angle of 173° and a laser wavelength of 633 nm (Zetasizer Nano-ZS, Malvern Instruments). Samples were prepared using a dilution of 1:10 (v/v) in Milli-Q water, and measurements were made in triplicate. The morphology of the NPs was analyzed by scanning transmission electron microscopy (STEM) (FESEM Ultra Plus) using a voltage of 20 kV and SE/InLens as detectors. For this purpose, a dilution of 1:100 (v/v) in Milli-Q water was stained with 2% (w/v) phosphotungstic acid and deposited on a copper grid, previously well-dried.

### 2.4 Stability of Blank Protamine NPs

The storage stability of blank NPs was determined for one month at 4 °C. The colloidal stability of blank 4:1 (w/w) Pr/Dx NPs was also measured in DMEM with/without 10% (v/v) of FBS and 1% (v/v) of P/S incubating at different time points: 0, 2, and 4 h at 37 °C under horizontal shaking (300 rpm). For both purposes, the size and PDI and DCR were determined by PCS using a dilution of 1:10 (v/v) in the corresponding media. The zeta potential was determined by LDA, as previously mentioned.

### 2.5 Nucleic Acid Association and Release

The 4:1 (w/w) Pr/Dx NPs were loaded with plasmid DNA (pDNA), miRNA, and siRNA at 8% (w/w) with respect to the theoretical total mass of solids. The genetic material was incorporated in the dextran solution used for NP formation. The morphology and physicochemical characteristics were determined as mentioned above. The nucleic acid association efficiency was determined by agarose gel electrophoresis at 1% (w/v) for pDNA and at 2% (w/v) for miRNA and siRNA using TAE buffer 1× as a running buffer. The nucleic acid-loaded NPs were incubated with an excess of heparin sodium salt at 1 mg/mL (25-fold with respect to the mass of nucleic acids) for 2 h at 37 °C. A maximum of 1 μg of genetic material (pDNA, miRNA, and siRNA) was loaded per line. The gel was run for 30 min in a Sub-Cell GT 96/192 (Bio-Rad Laboratories Ltd.) at 90 V. The signal of the SYBRGold 1× (Heidolph, Titramax 1000) was visualized using the Molecular Imager Gel Doc XR+ System (UV light 302; Bio-Rad) which determined the signal of the SYBR Gold 1× (Heidolph, Titramax 1000).

### 2.6 Cell Culture

The U87MG cell line was obtained from the American Type Culture Collection (ATCC). They were cultured in high DMEM supplemented with 10% (v/v) of FBS and 1% (v/v) of P/S at 37 °C with 5% of CO_2_ and 95% of relative humidity (Memmert INCO 2). The three patient-derived glioblastoma cell lines used in this study were GIN-8, GIN-28, and GCE-28. The project was approved by the National Research Ethics Committee East Midlands with Reference Number: 11/EM/0076. The GIN-8 cell line (Glioma INvasive margin cells) was isolated from the medial front invasive margin, the GIN-28 cell line was isolated from the 5-ALA fluorescence-positive invasive margin, and the GCE-28 cell line was isolated from the proliferative tumor core (Table S1).^23^ These three primary patient-derived glioblastoma cells were cultured in low DMEM supplemented with 10% (v/v) of FBS and 1% (v/v) of P/S at 37 °C with 5% of CO_2_ and 95% of relative humidity (Cryofusion, MCO_2_ OAIC-PE).

#### 2.6.1 Generation of Glioblastoma Spheroids

For the U87MG cell line, 250 cells/well were used to form the spheroids, whereas 2,000 cells/well were used for GIN and GCE cell lines. Cells were seeded in 96-ultra-low attachment round-bottom plates (ULA 96-well) in a final volume of 0.2 mL of supplemented DMEM. For the U87MG cell line, the cells were centrifuged for 20–30 min at 22 °C at 200 RCF and for patient-derived cell lines, 10 min at 300 RCF.

Morphological characterization of U87MG spheroids was carried out every 3–4 days by taking photos with an optical microscope (Olympus IX51, 10× magnification). Their size was determined by using the Olympus cellSens Standard Software. Spheroids between 200 and 300 μm were used for the experiments. Moreover, the morphology of U87MG spheroids was analyzed by scanning electron microscopy (SEM) (FESEM Ultra Plus, ZEISS). After the fourth day, the spheroids were fixed with 0.150 mL of commercial 10% (v/v) neutral buffered formalin and they were incubated for 15 min under horizontal shaking (Rocker) at RT, after washing with PBS 1× buffer. Then, the spheroids were carefully washed twice with PBS 1× buffer and once with Milli-Q water to initiate the dehydration process, where they were transferred in different dilutions of ethanol solution (20%, 50%, 70%, 90%, and 100% (v/v)). The dehydrated spheroids were deposited on a copper grid and analyzed by SEM using a voltage of 20 kV and SE/InLens (magnifications: 1.00, 3.00, 5.00, 10.00, and 30.00 KX). For GIN-8, GIN-28, and GCE-28 spheroids, morphological characterization was carried out after 2 days by taking photos using a plate reading widefield microscope (Nikon Intensilight C-HGFI/C-HGFIE, 10× magnification), where their size was determined using the NIS-Elements Viewer 5.21 software until reaching dimensions like those of U87MG spheroids.

### 2.7 Cytotoxicity Assay in 2D/3D Glioblastoma

The *in vitro* cytotoxicity of blank 4:1 (w/w) Pr/Dx NPs was first evaluated in a commercial U87MG cell line, followed by studies in primary patient-derived glioblastoma cell lines (GIN-8, GIN-28, and GCE-28) by the resazurin viability assay.

In the 2D cytotoxicity assay, 5 × 10^3^ cells were seeded in 96-well plates in a final volume of 0.1 mL of supplemented DMEM. After 24 h, the medium was replaced with 0.09 mL of fresh supplemented DMEM and 0.01 mL of (i) sterile filtered Milli-Q water (negative control), (ii) 1% (v/v) of Triton-X100 (positive control), and (iii) increasing doses of blank 4:1 (w/w) Pr/Dx NPs from 50 to 160 μg/mL. In 3D cytotoxicity assay, 0.150 mL of cell culture medium was carefully removed without sweeping along the spheroids. Different concentrations of the formulation were tested diluted in 0.150 mL of fresh DMEM supplemented medium (from 5 to 40 μg/mL in U87MG spheroids and from 5 to 80 μg/mL in GIN and GCE spheroids). The cells and spheroids were incubated with the formulation for 4 h at 37 °C. After the incubation time, glioblastoma cells and spheroids were washed and incubated in fresh supplemented DMEM for 24 and 48 h at 37 °C. To evaluate the NP cytotoxicity, the U87MG cells and spheroids were treated adding 0.02 and 0.04 mL of CellTiter Blue Cell Viability reagent, respectively, and GIN and GCE cells and spheroids were treated with 0.1 and 0.2 mL of 10% (v/v) of PrestoBlue Cell Viability reagent diluted in DMEM-free red phenol supplemented with 10% (v/v) of FBS and 1% (v/v) l-glutamine. The U87MG cells were incubated for 3 h, GIN and GCE cells were incubated for 2 h, and glioblastoma spheroids were incubated for 4 h in darkness at 37 °C. To measure the fluorescence emitted by the reduced form of resazurin in U87MG cells and spheroids, the reaction was stopped and stabilized by the addition of 0.05 mL of SDS 3% (w/v) for 30 min at 37 °C. The fluorescence signal was measured at 539 nm of excitation wavelength (λ_Ex_) and 620 nm of emission wavelength (λ_Em_) in a Synergy H1 microplate reader (Biotek) by Gen 5 software with their previous placement in black 96-well plates (BrandPlates pure grade). In the case of primary glioblastoma cells and spheroids, the fluorescence signal was measured at 544/590 nm (λ_Ex_/λ_Em_) on a BMG Labtech FLUOstar Omega microplate reader (Isogen Life Science B.V.) by Omega Software with previous placement in black 96-well plates (NUNCTM MicroWellTM).

The cell viability (%) was calculated as follows

$$cell\;viability\;\left(\%\right)=\frac{sample\;fluorescence}{control\;fluorescence}\times 100$$

Additionally, two complementary experiments were performed to achieve a complete 3D viability assay.

#### 2.7.1 Volume Assay of Glioblastoma Spheroids

The volume of glioblastoma spheroids was also analyzed before and after treatment with blank 4:1 (w/w) Pr/Dx NPs. Photos of U87MG spheroids were taken using an optical microscope (Olympus XI51, magnification 10×) and analyzed by Olympus cellSens Standard Software. Photos of patient-derived glioblastoma spheroids were taken using a plate reading widefield microscope (Nikon Intensilight C-HGFI/C-HGFIE, magnification 10×) and analyzed by NIS-Elements Viewer 5.21 Software. The area of the spheroids was measured using Fiji Software (ImageJ) and their volume (%) compared to the control was calculated as follows

$$spheroid\;volume\;\left(\%\right)=\frac{sample\;spheroid\;volume\;\left(*\right)}{control\;spheroid\;volume}\times 100$$

$$\left(*\right)\;spheroid\;volume=\frac{4}{3}\pi r^{3}\left({*}^{*}\right)$$

$$\left({*}^{*}\right)\;spheroid\;radius=\sqrt{\frac{area}{4\pi }}$$

#### 2.7.2 Membrane Integrity of U87MG Spheroids

This study was carried out under the same conditions as the 3D cytotoxicity assay. At selected time points after NP removal (4, 24, and 48 h), 0.005 mL of 7-AAD viability reagent was added directly in 0.2 mL of fresh supplemented DMEM. The plate was shaken gently by hand for 10 s, and the U87MG spheroids were incubated for 30 min at 37 °C in darkness. The 7-AAD expression was analyzed by fluorescence microscopy (Olympus XI51) using the mCherry channel by the Olympus cellSens Standard Software. In addition, the quantification of the fluorescence signal of 7-AAD was measured in a microplate reader (Synergy H1 microplate reader, λ_Ex_/λ_Em_ = 550/650 nm) by Gen 5 Software with the previous placement of the spheroids in black 96-well plates (BrandPlates pure grade).

Membrane integrity (%) was calculated as follows

$$spheroid\;membrane\;integrity\;\left(\%\right)=\frac{sample\;spheroid\;fluorescence}{control\;spheroid\;fluorescence}\times 100$$

### 2.8 NP Uptake Assay in 2D/3D Glioblastoma Models

#### 2.8.1 Polymer Labeling

To study NP internalization, protamine was labeled with the fluorescent reagent 5-TAMRA. For this purpose, protamine sulfate salt was dissolved in 0.1 M NaHCO_3_ buffer (pH = 8.58) at 10 mg/mL and 5-TAMRA was dissolved in DMSO at 10 mg/mL. After that, 0.06 mL of 5-TAMRA solution was added into 1 mL of protamine solution under mild stirring conditions (300 rpm) for 1 h at RT, resulting in complete homogenization. The magenta solution of 5-TAMRA-labeled protamine (Pr-TAMRA) was dialyzed using a cellulose membrane (Mw = 3.5 kDa, 16 mm dry, I.D. 35 feet, SnakeSkin) in 0.05 M NaCl buffer for 48 h and then in HPLC-grade water for 24 h under stirring conditions (500 rpm, RT, in the dark). Finally, the dialyzed solution was completed with HPLC-grade water until a final concentration of 5 mg/mL and lyophilized. The lyophilized product was stored in a desiccator.

The total amount of Pr-TAMRA was calculated as follows

$$mg\left(5‐TAMRA‐\mathrm{Pr}\right)=\frac{mg\left(vial+lyophilized\;product\right)}{mg\left(empty\;vial\right)}$$

#### 2.8.2 Formulation of 5-TAMRA-Labeled Protamine NPs

The formulation of NPs using TAMRA-labeled protamine was carried out following the protocol described in section “Formulation of Protamine NPs”. In this case, 0.5 mL of protamine solution was composed of 0.3 mL of Pr-TAMRA at 0.8 mg/mL and 0.2 mL of protamine at 3.8 mg/mL. The physicochemical characterization of the formulation was also done by triplicate measuring the particle size, PDI, DCR, and zeta potential under the same conditions mentioned in “Morphological and Physicochemical Characterization”.

#### 2.8.3 *In Vitro* 2D and 3D Uptake Assay

The internalization study of fluorescently labeled NPs in glioblastoma cells and spheroid cells was evaluated by confocal scanning laser microscopy (CSLM) (Leica TCS SP5 X, Leica Microsystems, GmB, and Leica CTR 6500, Leica Microsystems, TSC/SPE), SEM (FESEM Ultra Plus, ZEISS), and light sheet fluorescence microscopy (LSFM) with particle tracking microrheology (OptoRheo), respectively. The quantification was determined by flow cytometry (BD Accuri C6 Flow Cytometer and ImageStream X MkII Imaging Flow Cytometer-Luminex).

For the 2D uptake, 4.5 × 10^4^ U87MG, GIN-8, GIN-28, and GCE-28 cells/well were seeded in 24-well plates, using 12 mm diameter glass round coverslips covered with poly-l-lysine. Cells were cultured in a final volume of 1 mL of supplemented DMEM for 48 h at 37 °C. After cell incubation and glioblastoma spheroid formation, the culture medium was replaced with 7 μg/cm^2^ of 4:1 (w/w) Pr-TAMRA/Dx NPs and incubated for 4 h at 37 °C. Untreated cells and spheroids were used as a negative control. After this time, the U87MG cell line was washed with PBS 1× buffer and GIN and GCE cell lines with DPBS 10× buffer with calcium and magnesium chloride. Then, glioblastoma cells were fixed using 0.350 mL of commercial 10% (v/v) neutral buffered formalin, and spheroids were fixed with 0.150 mL of formalin for 15 and 30 min under horizontal shaking (Rocker) at RT, respectively. A 1:1000 dilution (v/v) of DAPI (stock concentration: 1 mg/mL in PBS 1×) was added and incubated for 30–45 min under the same conditions. After washing, the glass round coverslips were placed on slides using Fluoromount aqueous mounting medium and the fixed spheroids were placed on a μ-Slide-8-well chamber (1.5 polymer coverslip, tissue culture-sterilized, Ibidi) using the LAS X Life Science Software (63× magnification in U87MG and 40× in GIN-8, GIN-28, and GCE-28 cells, 20×-z2 magnification in U87MG and 10×-z1.5 in GIN and GCE spheroids, λ_Ex_/λ_Em_ (DAPI) = 358/461 nm and λ_Ex_/λ_Em_ (5-TAMRA) = 543/578 nm). In addition, the uptake in patient-derived spheroids was also analyzed by LSFM using OptoRheo^24^ in collaboration with Optics and Photonics Research Group (University of Nottingham). In this case, the fixed spheroids were placed on a μ-Slide-4-well chamber using a glass cube to reduce the size of the well adding up the spheroid in the center and aligned with the laser incidence.^25^ The TAMRA fluorescence signal was measured at 543/578 nm (60× magnification), and the images were processed by Fiji Software (ImageJ). Finally, the preparation of U87MG spheroids to analyze the NP uptake by SEM was carried out following the same protocol previously described in “Generation of Glioblastoma Spheroids”. Additionally, the internalization of 4:1 (w/w) Pr/Dx NPs loaded with siRNA labeled fluorescently with Cy5 (Cy5-siRNA) in the U87MG cell line was also evaluated by CSLM. The concentration of 8% (w/w) of Cy5-siRNA was associated with the NPs under RNase-free conditions. After the treatment of Cy5-siRNA-loaded NPs (7 μg/cm^2^, 4 h, 37 °C), the U87MG cells and spheroids were visualized by confocal microscopy (63× and 20× magnification, λ_Ex_/λ_Em_ (Cy5) = 635/670 nm) under the previously mentioned fixing conditions.

Flow cytometry was carried out to quantify the internalization of Pr/Dx NPs. In this case, after the same NP treatment to cells and spheroids (7 μg/cm^2^, 4 h, 37 °C), 0.2 mL of LIVE/DEAD Fixable Aqua Dead Cell Stain reagent diluted in PBS 1× buffer was added in glioblastoma cells, 0.01 mL of this reagent was added to 0.05 mL of the spheroid suspension, and they were incubated for 15 and 30 min in horizontal shaking at RT and 37 °C, respectively. After washing, the spheroids were collected in a 15 mL conical falcon tube for their settlement for 2 min at RT. The culture medium was replaced carefully by PBS 1× buffer in U87MG spheroids and by DPBS 10× buffer in GIN and GCE spheroids, and they were centrifuged (Centrifuge 5430R, Eppendorf, HAWK 15/05 refrigerated centrifuge, Sanyo MSE, respectively) for 1 min, 200 RCF at 22 °C. The glioblastoma cells were detached, and the spheroids were deaggregated with 0.05% Trypsin 1×-EDTA for 5 and 20 min at 37 °C, respectively. In the case of the spheroids, manual pipetting was used to aid the deaggregation of the spheroids. After trypsin deactivation by adding supplemented DMEM, the cells were transferred to Eppendorf tubes and centrifuged for 5 min (Centrifuge 5430R and HAWK 15/05 refrigerated centrifuge). In the case of the U87MG cell line, the pellet was resuspended in 0.5 mL of PBS 1× buffer supplemented with 10% (v/v) of FBS, and in the case of the patient-derived GIN and GCE cell line, the pellet was resuspended in 0.05 mL of commercial 4% (v/v) paraformaldehyde. Finally, a maximum of 10,000 U87MG events were excited at 488 nm using filters BP 575/25 for 5-TAMRA and at 405 nm using filters BP 515/20 for Aqua viability reagent, and they were analyzed by BD CSample Software (BD Biosciences). The same amount of GIN and GCE events was excited at 561 nm for 5-TAMRA and at 405 nm for Aqua viability reagent, and they were analyzed by IDEAS 6.2 Software (Luminex).

### 2.9 Transfection Assay in 2D/3D Glioblastoma Models

#### 2.9.1 Amplification of Plasmid DNA

To perform the transfection studies, a plasmid encoding the Enhanced Green Fluorescence Protein (EGFP) and Luciferase protein (Luc) (pEGFP-Luc) was selected. The plasmid pEGFP-Luc were amplified using competent DH5α bacteria following the standard protocol described elsewhere.^26^ The plasmid extraction was performed using the Invitrogen HiPure Plasmid Gigaprep Kit following the manufacturer’s protocol. For this purpose, the transformed bacteria were ultracentrifuged (Avanti J-26 XPI Centrifuge, rotor JA-10, 10,000 rpm) for 15 min at 4,000 RCF. The bacteria pellet was resuspended in RNase solution and mixed with a lysis buffer. The removal of cell debris was carried out using a precipitation buffer, and the plasmid was purified using the columns provided by the kit. Finally, the plasmid was eluted and precipitated, and the concentration was quantified by spectral scanning UV–VIS spectrophotometry (NanoDrop). The concentration was calculated by measuring the pure plasmid solution and different dilutions. The measurements were done in triplicate.

#### 2.9.2 *In Vitro* 2D/3D Transfection Assay

The transfection studies using 4:1 (w/w) Pr/Dx NPs in U87MG glioblastoma cells and spheroids were carried out using plasmid pEGFP-Luc. As mentioned above, 8% (w/w) of pEGFP-Luc with respect to the total mass of solids were associated with the NPs. For the 2D transfection assay, 4.5 × 10^4^ glioblastoma cells/well were seeded in 24-well plates in a final volume of 1 mL of DMEM supplemented medium and were incubated for 24 h at 37 °C. After cell incubation and spheroid formation, the U87MG cells and spheroids were treated with (i) different concentrations of NPs corresponding to 0.5, 1, and 2.5 μg of pDNA/well, (ii) naked pEGFP-Luc (negative control), and (iii) Lipofectamine 2000 reagent, prepared under the specifications of the commercial protocol (positive control). All groups were prepared in 0.2 mL of Opti-MEM non-supplemented culture medium. The formulation and controls were incubated for 4 h at 37 °C. The evaluation of EGFP expression 24 and 48 h after NP removal was carried out by direct observation using a fluorescence microscope (Olympus IX51) and Olympus cellSens Standard Software.

Additionally, the capacity of the NPs to deliver the therapeutic miRNA-145 was evaluated in the U87MG cancer cell line. For this purpose, 2.5 × 10^5^ glioblastoma cells/well were seeded in a 6-well plate in a final volume of 2 mL of DMEM supplemented medium and were incubated for 24 h at 37 °C. Then, the corresponding amount of 4:1 (w/w) Pr/Dx NPs for 5 μg of miRNA-145 was added in a final volume of 1 mL. Cells transfected with the same amount of NPs loaded with the scrambled sequence were the control. After 4 h of incubation, the medium was replaced with fresh DMEM supplemented medium in a final volume of 2 mL. The transfection efficiency was determined after 48 h by Real Time-PCR (Strategene Mx 3000, Agilent Technologies). Briefly, the total miRNA was extracted from U87MG cells using the MicroRNA Purification kit. miRNA concentration and purity was determined with UV spectrophotometry (NanoDrop, Spectrophotometer ND-1000). cDNA synthesis was carried out from 120 ng of total miRNA with the qScriptTM microRNA cDNA synthesis kit. Quantitative RT-PCR was carried out using PerfeCta MicroRNA Assays, with a primer for miR145 (has-miR145-5p). Small nuclear RNA, RNU6, was used as an internal control for miRNA expression (5′CTCGCTTCGGCAGCACA3′; 5′AACGCTTCACGAATTTGCGT 3′). Each PCR cycle consisted in 2 min at 95 °C activation, 5 s at 95 °C denaturation, and 30 s at 60 °C annealing (40 cycles). Quantitative data were analyzed by using MxPro software. Relative expression levels of miRNA-145 in each treatment group were calculated by the delta*Ct* method, as described by Livak and Schmittgen^27^ in relation to RNU6 levels and normalized with respect to untreated control cells.

### 2.10 Zebrafish Care and Maintenance

Adult zebrafish (Danio rerio, WT) were maintained in 30 L aquaria with a ratio of one fish per liter of water, a 14:10 light/night cycle, and a mean temperature of 28.5 °C according to the procedures described elsewhere.^28^ Zebrafish embryos were obtained by mating the adults under controlled conditions. All procedures used in the experiments, fish care, and treatment were performed in agreement with the Animal Care and Use Committee of the University of Santiago de Compostela (project reference: 01/20/LU-003) and the standard protocols of Spain (Directive 2012-63-UE) and Spanish Government guidelines (Real Decreto 53/2013), conducted in the animal facilities in the Veterinary School of the University of Santiago de Compostela (Campus Lugo) (AE-LU-003). At the final point of the experiments, zebrafish embryos were euthanized by tricaine overdose.

### 2.11 Fish Embryo Acute Toxicity Test

The toxicity assay in WT zebrafish embryos was carried out following the official Fish Embryo Acute Toxicity test (FET) of the Organization for Economic Cooperation and Development (OECD 203).^21^ At least three replicates were performed; for each one, 10 embryos (30 embryos in total) were treated with increasing NP concentration, from 0.5 to 3.5 μg/mL, and 10 embryos were treated with osmosis water as a negative toxicity control and with 3,4-dichloroaniline as a positive toxicity control. Embryos were inspected under an inverted optical microscope (Nikon TMS) at 24, 48, 72, and 96 h of treatment. To determine the embryo lethality, the microscope observations were focused on coagulation of embryos, lack of somite formation and nondetachment of the tail, lack of heartbeat after, and hatching rates according to the FET test indications. Development alterations and embryo malformations were also recorded. To be a valid test, the mortality of fish embryos in the negative control after 96 h must not exceed 10% and the mortality in the positive control must be at least 30%.

### 2.12 Statistical Analysis

Differences were statistically analyzed by one/two-way ANOVA followed by Tukey’s method, respectively, if not stated otherwise. All statistical analyses were conducted using GraphPad Prism Software (version 8.0 for Windows). A *p* < 0.05 was considered significant (*p < 0.05; **p < 0.01; ***p < 0.001; ****p < 0.0001). Each experiment was performed independently in triplicate (*n* = 3), if not stated otherwise.

## 3 Results and Discussion

### 3.1 Physicochemical Characterization of Protamine NPs

An ionic inter-complexation method was used to formulate protamine NPs. Briefly, the dextran solution was added to the protamine solution, allowing the spontaneous formation of the NPs through electrostatic interactions. Parameters such as the ratio of materials, the interaction mechanisms, and the processing conditions play an important role in formulating optimal nanocarriers with maximum therapeutic efficacy. This method based on cross-linking positively charged molecules with negatively charged polyanions presents several advantages such as the use of water-based solutions, mild reaction conditions, simplicity, and cost-effectiveness.^29^ Protamine is a polycationic protein and was selected as a material for the NPs due to its capacity to condense nucleic acids and its cell permeation properties. Dextran sulfate is a polyanionic polysaccharide and was selected for its gelling capacity.^9,10,13^ Based on previous screening studies carried out by our research group,^30^ a mass ratio 4:1 (w/w) Pr/Dx was selected for NP formulation as it leads to the best characteristics regarding association, protection, and intracellular transport of genetic material. In the process of forming nucleic-acid-loaded NPs, the nucleic acid was directly incorporated into dextran solution before NP formation, ensuring efficient encapsulation without compromising the structure or stability of the nanosystem. Regarding the loading of genetic material in this formulation, an 8% (w/w) payload with respect to the theorical total mass of NPs was selected. This loading percentage represented the maximum amount of nucleic acid encapsulated without compromising the morphology and physicochemical properties of the NPs, such as size, formulation homogeneity, and surface charge.^30^ Maintaining these properties is crucial for ensuring the stability and efficacy of the NPs in gene delivery applications.

In this work, the NPs were characterized for mean particle size, PDI, surface charge, and morphology. In all cases, the formulation was composed of a homogeneous NP population (PDI ≤ 0.2) with spherical morphology (Figure 1A), indicating uniformity in particle size, which is essential for consistent biological interactions. An average size between 100 and 200 nm is suitable for preventing rapid renal excretion and promoting a prolonged circulation time *in vivo*. Additionally, this size favors enhanced permeability and retention (EPR) effects, which are advantageous for tumor targeting. The positive surface charge reflects the cationic nature of protamine, which facilitates strong electrostatic interactions with negatively charged cell membranes, enhancing the cellular uptake. Moreover, the high zeta potential values suggest that the NPs possess adequate electrostatic repulsion to remain stable in suspension, reducing the likelihood of aggregation over time. This is crucial for maintaining their bioavailability and therapeutic efficacy *in vivo* (Table 2).^22,31,32^

**Figure 1:**
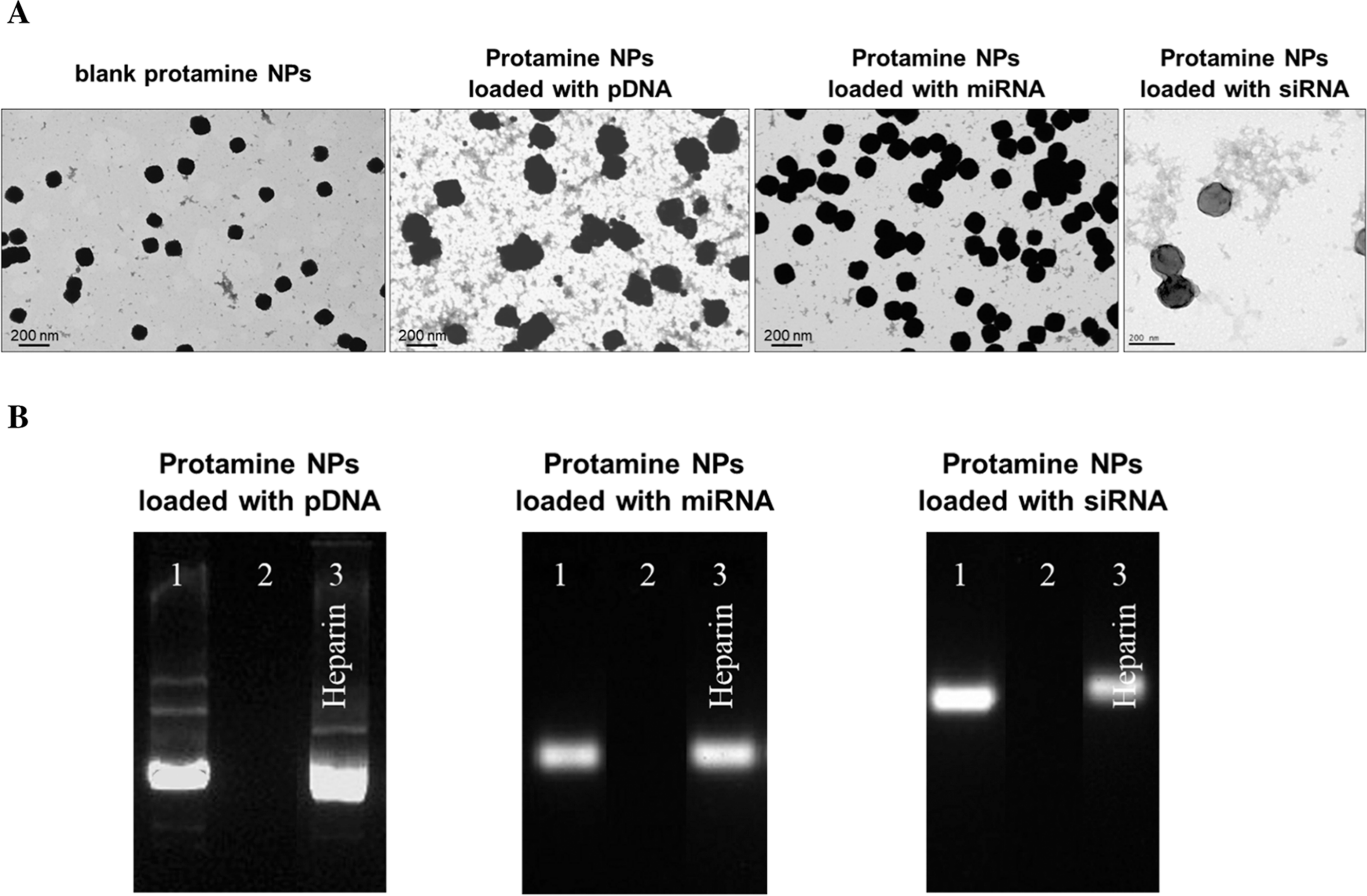
Physicochemical characterization of nucleic acid-loaded protamine NPs. STEM images of blank 4:1 (w/w) Pr/Dx NPs and loaded with pDNA, miRNA, and siRNA (A). Images from agarose gel electrophoresis of 4:1 (w/w) Pr/Dx NPs loaded with 8% (w/w) of pDNA, miRNA, and siRNA. Lane 1: naked pDNA, miRNA, siRNA. Lane 2: NPs at *t* = 0 h. Lane 3: displacement assay where the NPs are incubated with heparin for 2 h at 37 °C (1:25 w/w nucleic acid/heparin ratio). The amount of nucleic acid per lane was 1 μg (B). Scale bar = 200 nm.

**Table 2:** Mean Particle Size, PDI, and Zeta Potential of 4:1 (w/w) Pr/Dx NPs, Blank, and Loaded with 8% (w/w) of pDNA, miRNA, and siRNA (Mean ± SD (*n* = 3))

4:1 (w/w) Pr/Dx NPs	Size (nm)	PDI	Zeta Potential (mV)
blank	120 ± 7	0.2	34 ± 2
pDNA	146 ± 1	0.2	33 ± 5
miRNA	124 ± 5	0.1	30 ± 3
siRNA	157 ± 16	0.1	18 ± 4

The association of the nucleic acids with the NPs was studied by agarose gel electrophoresis. The results showed an effective binding of pDNA, miRNA, and siRNA (Figure 1B, lanes 2). The fact that free nucleic acids were not detected demonstrates the capacity of our NP prototype to bind efficiently different nucleic acids. To explore whether this binding is reversible, the formulations were incubated with an excess of heparin, a highly charged glycosaminoglycan that can cause the displacement of the nucleic acids from the NPs^33^ (Figure 1B, lanes 3). The observation of the band under these conditions confirms that nucleic acids are loaded in the formulations and that they could be released under adequate circumstances.

### 3.2 Storage of Blank Protamine NPs

NPs can change their critical properties under storage. Thus, the optimization of the storage conditions is important to preserve their physical characteristics critical for their performance.^34^ In the present work, the stability of blank Pr/Dx NPs under storage conditions was studied by monitoring mean particle size, PDI, surface charge, and DCR of the formulation in aqueous suspension for 30 days at 4 °C (Figure S1A). After this time, the formulation experienced an increase in particle size over time, but was constantly below 150 nm without increasing its PDI. In addition, the DCR was maintained within the same range throughout, indicating the absence of significant aggregation phenomena. The NPs maintained a positive zeta potential above +40 mV over the studied period. This colloidal stability profile is consistent with the stability data reported for other protamine nanosystems previously studied, such as protamine hyaluronic acid NPs and protamine nanocapsules.^9,10,22^

### 3.3 Colloidal Stability of Blank Protamine NPs

The stability of this formulation was also measured when it was suspended in cell culture media at different time points. This test was important to determine their feasibility for *in vitro* testing and potential stability in biological media. The results collected indicated that blank Pr/Dx NPs diluted in supplemented DMEM maintained their particle size, even after long incubation times. However, particle size increased in non-supplemented DMEM (Figure S1B), together with PDI values. PDI values larger than 0.4 describe a polydisperse nanosystem, indicative of instability.^35^ This was corroborated by a decrease in the DCR, especially when Pr/Dx NPs were diluted and incubated in non-supplemented DMEM for 4 h, which indicates sedimentation of particle aggregates. We suggest that NP electrostatic repulsion is masked by changes in pH or the ionic strength of the cellular medium, leading to aggregation. However, this process could be controlled by FBS supplementation, suggesting that protein adsorption on the surface of NPs could provide a stabilization mechanism. Therefore, these results indicated that cell culture conditions using supplemented DMEM were favorable to perform *in vitro* studies with these NPs.

### 3.4 Morphological Characterization of Glioblastoma Tumor Spheroids

A 3D spheroid model was used to complement the results from monolayer cell cultures and better reflect the pathophysiological environment in terms of cell–cell interactions, nutrients, and oxygen gradients and could be used to assess toxicity effects and NP uptake.^36^

Spheroids were prepared from U87MG cells (250 cells/spheroid) and from three primary patient-derived cells: GIN-8, GIN-28, and GCE-28 cells (2000 cells/spheroid). The spheroid size is a critical parameter related to tumor biology and is determined by three factors: cell type, culture time, and seeding density. Considering this information, the spheroids were cultured at standard conditions to obtain a size between 200 and 300 μm. Previous research by our group identified small glioblastoma spheroids, approximately 250 μm in diameter, as optimal 3D culture models for *in vitro* studies. This specific size was chosen due to factors such as the limited penetration of excitation wavelengths in microscopy and the potential onset of necrosis within the spheroid core. Cells at the core of the spheroids are prone to necrosis due to hypoxic conditions and nutrient deprivation, which presents a challenge for efficient gene delivery.^37^ However, necrosis is a physiologically relevant characteristic of primary glioblastoma tumors. Therefore, these transport limitations are directly relevant to the model, providing a more accurate simulation of tumor conditions, and thereby increasing the value of these studies.

In the present work, at day 4 post-cell seeding, the U87MG spheroids reached the appropriate size (250 μm) and morphology, while the primary patient-derived cells formed 250 μm spheroids by day 2 (Figure 2). Some differences between the morphology of the spheroids were observed, especially those formed by patient-derived glioblastoma cell lines. For example, GIN-28 and GCE-28 cells formed more compact spheroids than the GIN-8 cell line (Figure 2A). This could be explained by the fact that the GIN-28 and GCE-28 were derived from intratumor regions (invasive margin and tumor core, respectively) from the same patient, whereas GIN-8 cells were obtained from another patient.^23^ In contrast, SEM images displayed in Figure 2B showed the establishment of close interactions among the cells leading to fully developed spheroids. In addition, some cell–cell gaps at the surface of the U87MG spheroid could be also observed, which was anticipated to be advantageous for NP internalization.^38^

**Figure 2:**
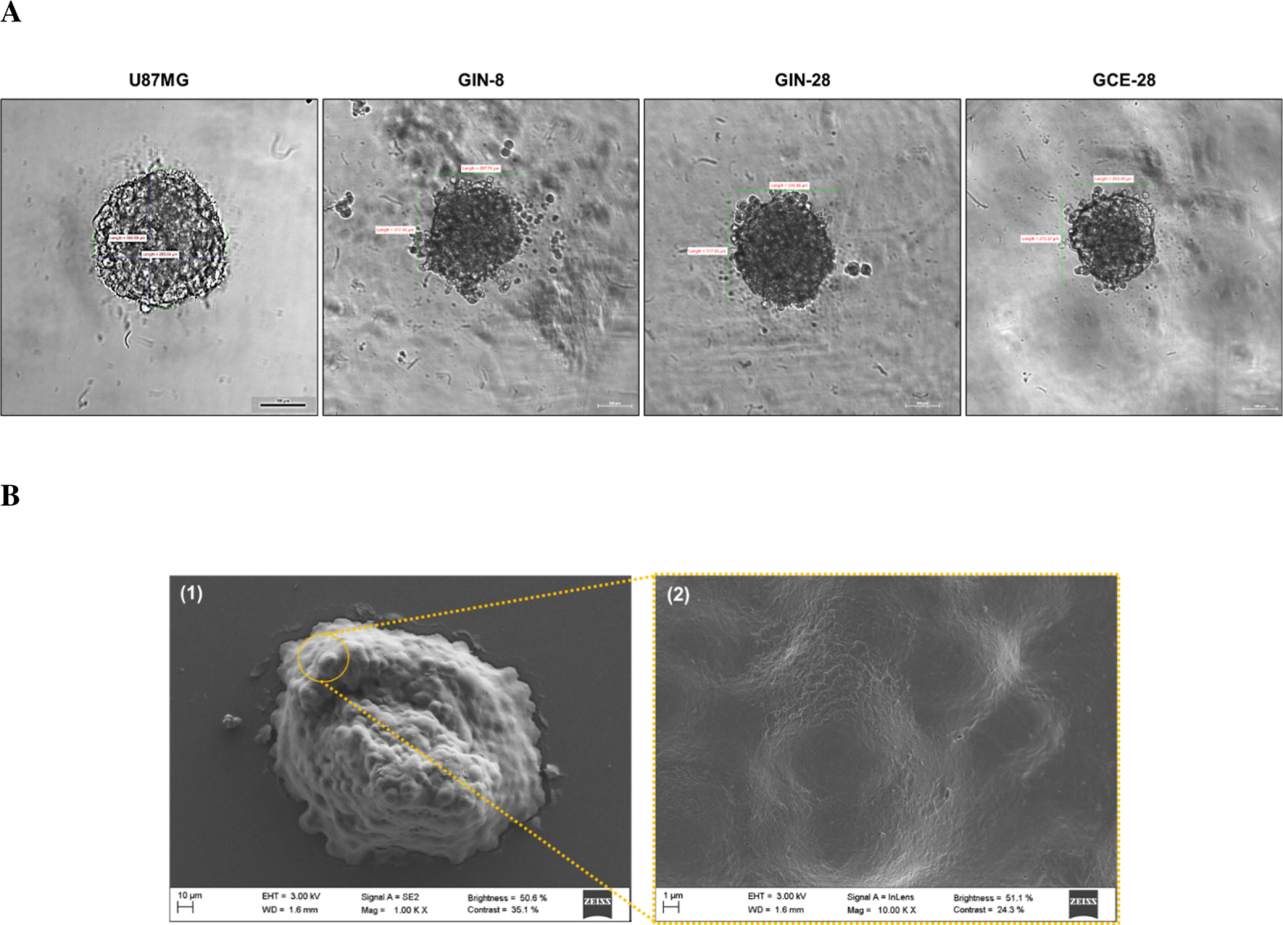
Phase-contrast microscopy images of U87MG spheroids on the 4^th^ day and GIN-8, GIN-28, and GCE-28 spheroids on the 2^nd^ day (magnification 10×, scale bar = 100 μm) (A). SEM images of U87MG spheroids on the 4^th^ day (B): morphology of the spheroids (1) and a detail of their cells (2) (scale bar = 10 and 1 μm, respectively).

### 3.5 Cytotoxicity Assessment of Protamine NPs

The main objective of this study was to evaluate the safety of this nanosystem in advanced preclinical models. This is an essential step in the early pharmaceutical development stages of gene delivery nanosystems as cytotoxicity is a major limiting factor in these therapies. For this purpose, 3D spheroid models provide a more informative model than standard flat cultures as they can better mimic the characteristics of their microenvironment.^19,20^ The cytotoxicity of NPs depends on their physicochemical properties, with an excess of positive charge on the NP surface possibly conferring increased toxicity.^39^ Therefore, we aimed to demonstrate the compatibility of Pr/Dx NPs with translationally relevant cell lines. To that purpose, cell viability was evaluated in a panel of primary cell lines derived from different glioblastoma regions: the invasive margin (GIN-8 and GIN-28) and tumor core of glioblastoma (GCE-28).^40^

As previously mentioned, low molecular weight protamine is a natural peptide that has the ability to penetrate cells without causing toxicity. This safety feature was confirmed in the present work, where no significant difference in cell viability was observed, indicating that our nanosystem did not exhibit cytotoxicity on U87MG, GIN, and GCE cell lines in monolayer (0.9965 (U87MG); 0.7946 (GIN-8); 0.8781 (GIN-28); and 0.9939 (GCE-28)) (Figure 3A) nor in 3D cell culture models (0.9260 (U87MG); 0.9694 (GIN-8); 0.9869 (GIN-28); and 0.9407 (GCE-28)) (Figure 3B). The cytotoxicity levels observed in this formulation are consistent with data reported in the literature about other polymeric NPs in which protamine was combined with different polysaccharides to dextran, such as hyaluronic acid and alginate.^22,41–43^ Moreover, Thomas et al. specifically demonstrated that dextran-protamine polycations mediated high *in vitro* transfection efficiency without significant cytotoxicity making them a promising alternative for gene delivery.^14^

**Figure 3:**
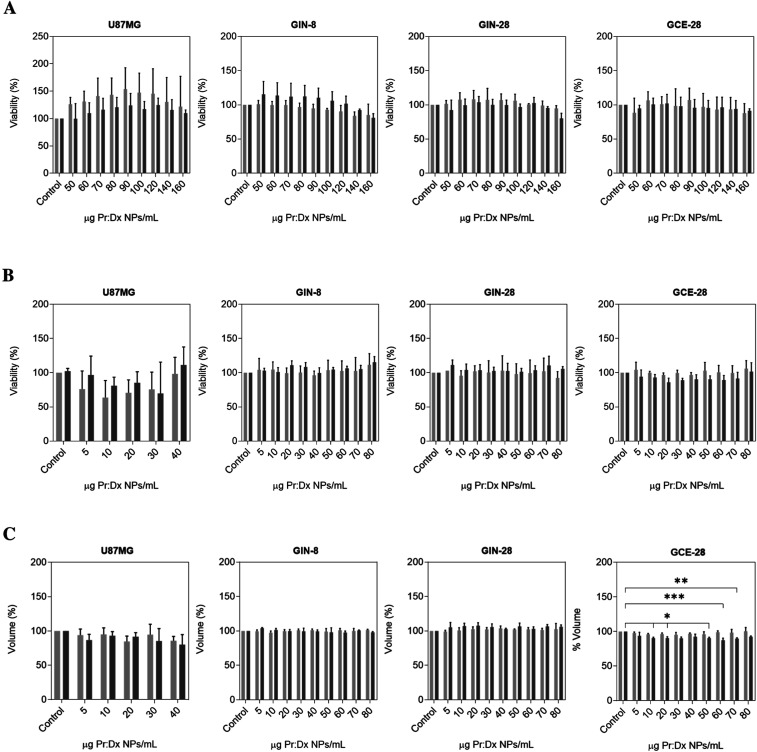
Cell metabolic activity assay of U87MG, GIN-8, GIN-28, and GCE-28 cells (A) and spheroids (B), and volume of U87MG and GIN-8, GIN-28, and GCE-28 spheroids (C) after 24 h (light-gray bars) and 48 h (dark-gray bars) of the removal of increasing concentrations of blank 4:1 (w/w) Pr/Dx NPs. Control: glioblastoma cells and spheroids treated with sterile filtered Milli-Q water (mean ± SD (*n* = 3)).

To perform a comprehensive 3D viability study, the volume and morphology of glioblastoma spheroids were also assessed. First, this parameter was qualitatively analyzed by phase-contrast microscopy images (Figure S2). A negligible reduction in the volume of the spheroids was observed for all cases, as compared with the positive control for cytotoxicity (1% (v/v) of Triton-X100), which caused a reduction in the spheroid size leading to its disintegration, indicating the sensitivity of the assay. In addition, the spheroids maintained a compact morphology after treatment; however, cellular extensions could be observed in GIN-8 and GIN-28 spheroids, which were more pronounced with time and at higher NP concentration. This cell dynamism is justified as both GIN cell lines are derived from the invasive region of glioblastoma, and may thereby retain migratory/invasive potential *in vitro*.^23,44,45^ Spheroid volume was quantified from the images by measuring the spheroid area (Figure 3C). In general, the data confirmed that blank Pr/Dx NPs did not cause a marked reduction in spheroid volume compared with the negative control for cytotoxicity, especially in GIN spheroids, where the spheroid volume remained constant. Indeed, GIN glioma cells have a more aggressive phenotype than GCE core cells, making them more resistant to therapy.^44,45^ In GCE-28 spheroids, the volume significantly decreased slightly at higher concentrations, especially 48 h post-treatment.

Another orthogonal viability assay was performed by analyzing the membrane integrity of the cells in the U87MG spheroids. For this purpose, the internalization of 7-AAD, a fluorescent apoptosis marker with a strong affinity for DNA, was measured. In this assay, dead cells exhibit red fluorescence, whereas living cells do not exhibit fluorescence. Spheroids treated with Triton X-100, as a positive control of cell toxicity, exhibited an intense red fluorescence signal, whereas spheroids treated with sterile filtered Milli-Q water, as a negative control of cytotoxicity, did not show this intense fluorescence. Spheroids treated with Pr/Dx NPs did also not show any 7-AAD fluorescence, which indicates no cytotoxicity under these experimental conditions (Figure S3). As a general conclusion, the results from these studies collectively demonstrate low cytotoxicity of these NPs, which is important for their intended use in glioblastoma treatment.

### 3.6 Cellular Uptake of Protamine NPs

To study the internalization of Pr/Dx NPs in glioblastoma cells, protamine was fluorescently labeled with 5-TAMRA, a succinimidyl ester displaying good reactivity and specificity toward primary and secondary aliphatic amines, resulting in the formation of stable amides that closely resemble natural peptide bonds.^46^ Among all amino acid residues forming the structure of protamine, the N-terminal proline residue is the most reactive toward this succinimidyl group.^47–49^ The formulation of Pr/Dx NPs using 5-TAMRA-labeled protamine displayed similar physicochemical characteristics to the non-labeled formulation. In addition, in order to track the intracellular delivery of the associated therapeutic biomolecules, this study was also performed with non-fluorescent NPs loaded with 8% (w/w) of a siRNA labeled with Cy5 (Table 3).

**Table 3:** Mean Particle Size, PDI, and Zeta Potential of 4:1 (w/w) Pr/Dx NPs Labeled with TAMRA and Nonfluorescent NPs Loaded with 8% (w/w) of Cy5-siRNA (Mean ± SD (*n* = 3))

4:1 (w/w) Pr/Dx NPs	Size (nm)	PDI	Zeta Potential (mV)
5-TAMRA Pr	131 ± 6	0.2	29 ± 2
Cy5-siRNA	196 ± 23	0.1	29 ± 1

NP internalization was analyzed by confocal microscopy 4 h after treatment with the TAMRA-labeled formulation. This includes both the control group (blank NPs) and the group loaded with a fluorescent nucleic acid. The images corresponding to the orthogonal sections on the *X* and *Y* axes of U87MG, GIN, and GCE cells and spheroids showed the intracellular localization of Pr/Dx NPs. These NPs were also associated with Cy5-siRNA, verifying their cellular internalization along with the associated biomolecules (Figure 4). The efficient intracellular internalization of Pr/Dx NPs was likely due to the penetration enhancing properties of protamine, as previously described by our research group for polymeric nanocapsules^9,10,50^ or in lipid–polymeric hybrid NPs.^51^ It has been reported that the intracellular delivery could also be increased by modifications on the NP surface by coating with protamine^52,53^ or by the NP functionalization with this peptide.^54,55^ Moreover, studies found that six consecutive arginine residues in the protamine structure constitute a nuclear localization signal (NLS), which would explain why fluorescence accumulated close to the cell nucleus.^56,57^

**Figure 4:**
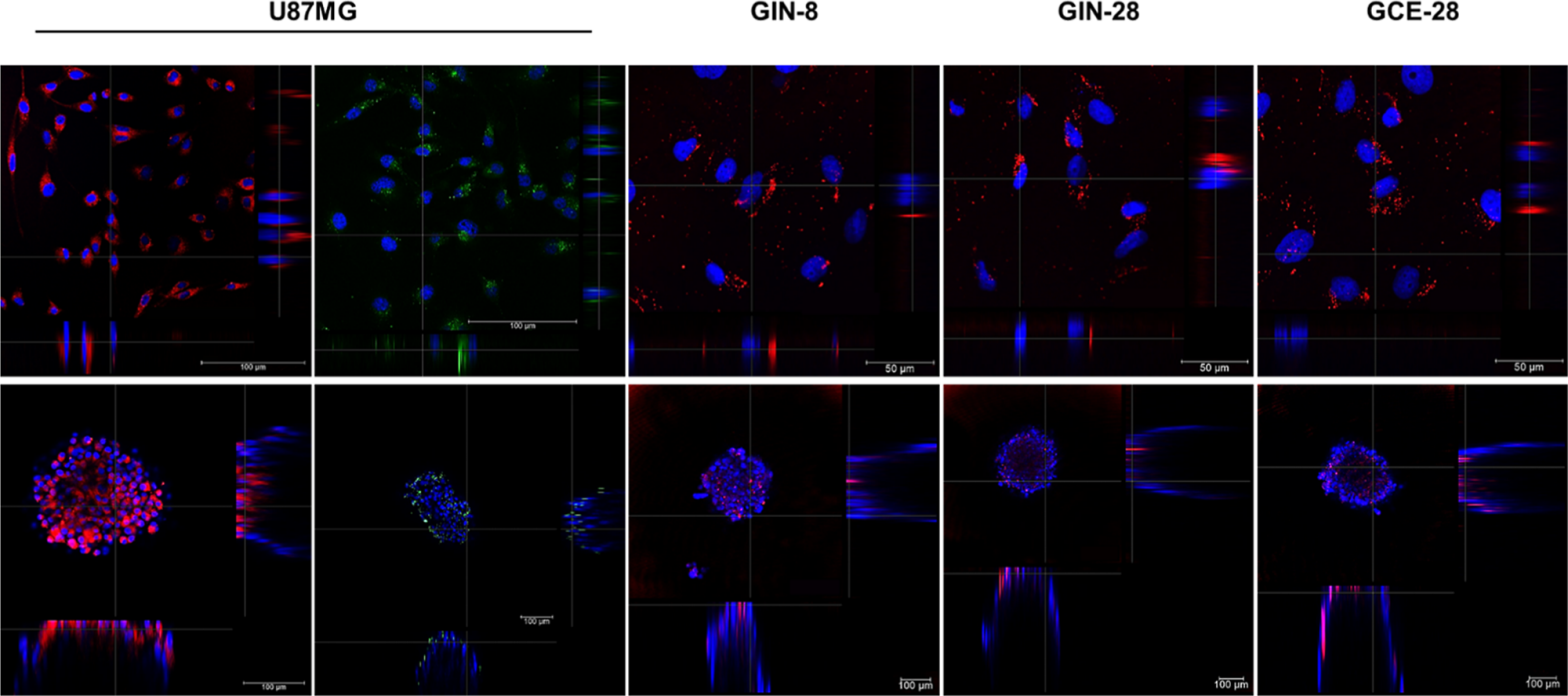
Maximum projections confocal microscopy images and orthogonal sections in cellular models. Top line: U87MG, GIN-8, GIN-28, and GCE-28 cells (magnification 63× and 40×, scale bar = 100 and 50 μm, respectively). Bottom line: U87MG, GIN-8, GIN-28, and GCE-28 spheroids (magnification 20× and 10×, z2 and z1.5, respectively). Cells and spheroids were treated with fluorescently labeled NPs (7 μg/cm^2^, red channel) and NPs loaded with 8% (w/w) of fluorescent Cy5-siRNA (7 μg/cm^2^, green channel). Nuclei of the cells were stained with DAPI (blue channel) (scale bar = 100 μm).

Despite obtaining good NP intracellular internalization in both *in vitro* models, we observed some differences regarding the distribution and localization of the NPs in the spheroids formed by established cell lines vs primary cell lines derived from patients. In addition to the physicochemical characteristics of the NPs, the cell type, preparation method, mean size/size distribution, and cellularity of the spheroids also play a crucial role in the NP spheroid interaction.^58,59^ In glioma cells, differences in the NP internalization can be linked to distinct endocytic pathways. Among the primary glioma cells, GIN cells often exhibit a higher rate of endocytosis due to their more active proliferation and enhanced metabolic demand, which drives an increase in NP internalization.^40^ However, U87MG spheroids displayed the highest intensity of fluorescence that was homogeneously distributed along the spheroid, suggesting better and more uniform distribution of protamine NPs (Figure 4).

SEM images showed how these Pr/Dx NPs were embedded by the U87MG spheroid (Figure 5A). Conversely, light sheet microscopy analysis of GIN and GCE spheroids revealed higher accumulation of fluorescent NPs close to the spheroid surface as compared to the core (Figure 5B).

**Figure 5:**
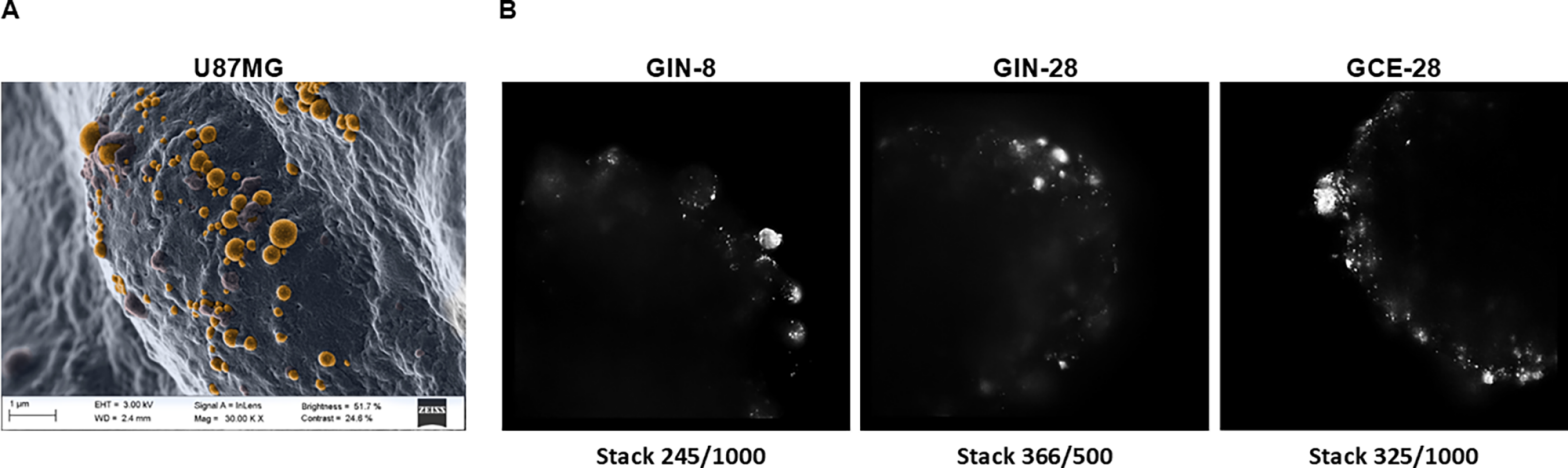
SEM image of U87MG spheroids treated with blank 4:1 (w/w) Pr/Dx NPs artificially colored in orange (7 μg/cm^2^, scale bar = 1 μm) (A). Light sheet fluorescence microscopy images (OptoRheo) of GIN-8, GIN-28, and GCE-28 spheroids treated with fluorescently labeled NPs (seen as bright-white spots, 7 μg/cm^2^, magnification 60×) (B).

To provide a quantitative evaluation, NP uptake was additionally assessed via flow cytometry after treating the cells and spheroids with the LIVE/DEAD Fixable Aqua Dead Cell Stain reagent. This allowed us to examine the uptake, specifically in living cells. In the case of spheroids, it was necessary to first disaggregate the spheroids to measure single fluorescent events. The flow cytometry histograms verified the internalization of blank Pr/Dx NPs in both 2D (Figure 6A) and 3D glioblastoma models (Figure 6B). More specifically, the shift of the peak to the right side indicated a higher fluorescence intensity. Compared to untreated cells (unstained controls), ∼100% of the U87MG, GIN, and GCE cells (Table S2) and cells forming the U87MG spheroids (Table S3) were positive for the presence of Pr/Dx NPs. In GIN and GCE spheroids, these values were significantly lower (GIN-8: 63%, GIN-28: 59%, and GCE-28: 57%) confirming the conclusions from the confocal image study (Table S3). Besides this, the peak corresponding to the fluorescent signal of Aqua was very intense in all cases in the negative (“live”) region, which confirms the low toxicity of the NPs in both 2D cultures (Figure S5A) and spheroids (Figure S5B) and the conclusion from the previous section. Additionally, in the primary patient-derived models, images obtained by the ImageStreamer flow cytometer also confirmed the presence of fluorescent intracellular NPs within cells and spheroids (yellow color), compared to images of the corresponding controls (Figure S6).

**Figure 6:**
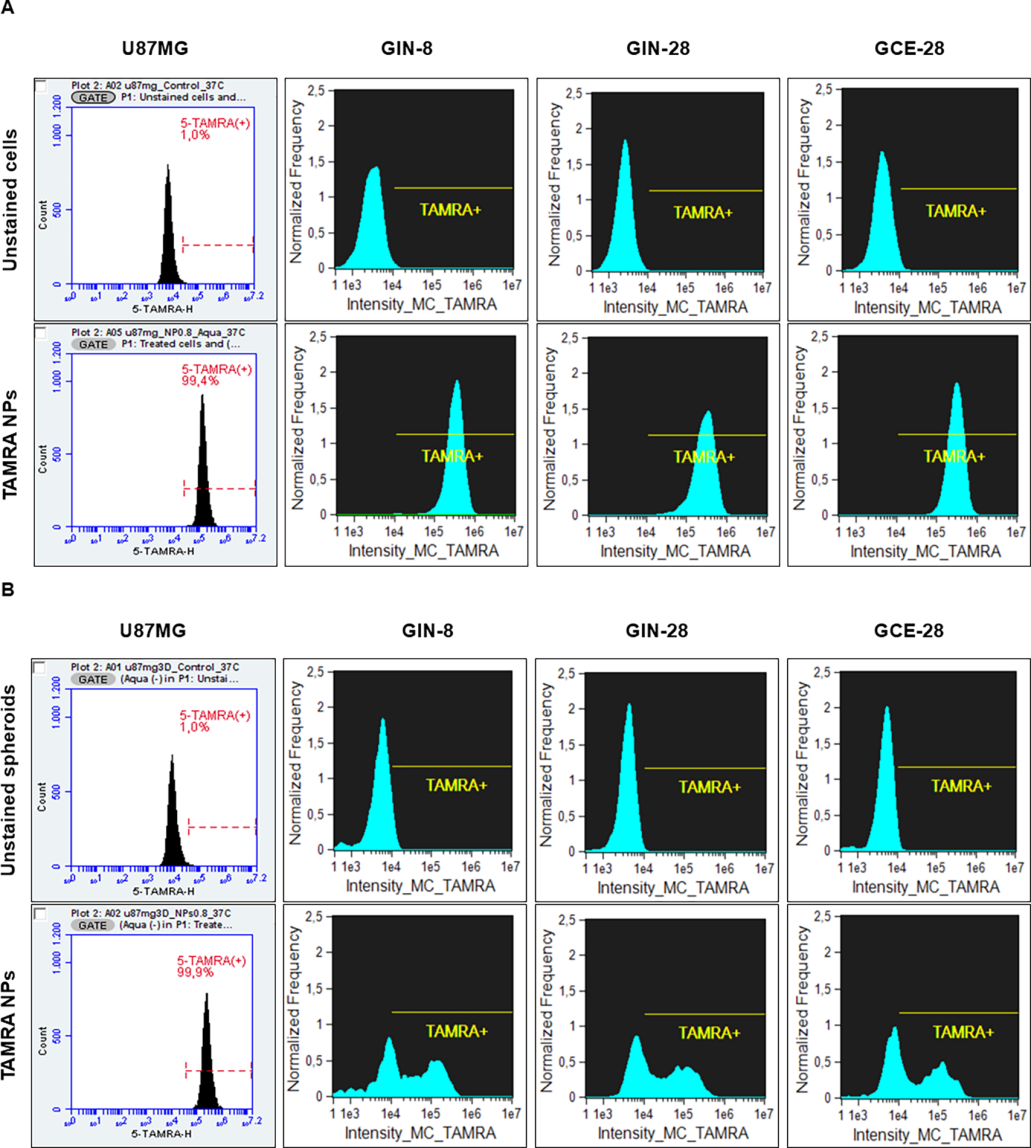
Flow cytometry histograms to quantify the total positive events (peak) of control, U87MG, GIN-8, GIN-28, and GCE-28 cells (A) and spheroids (B) treated with fluorescently labeled TAMRA-NPs (7 μg/cm^2^) after 4 h post-treatment.

### 3.7 Transfection Capacity of Protamine NPs

The next step involved the evaluation of Pr/Dx NP transfection efficacy to demonstrate the potential of these NPs as a gene delivery system. The transfection capacity of the NPs was evaluated in both 2D and 3D *in vitro* models, by analyzing the expression of the EGFP protein upon exposure to NPs loaded with an EGFP-plasmid. The fluorescence microscopy images revealed expression of this protein after 24 and 48 h of the incubation of the NPs with U87MG cells and spheroids (Figure 7). Even though different pDNA doses were evaluated, there was no improvement in the transfection efficiency for doses exceeding 1 μg/well. These observations confirm positive results obtained with several other protamine-based gene nanocarriers.^9,10,47,60^

**Figure 7:**
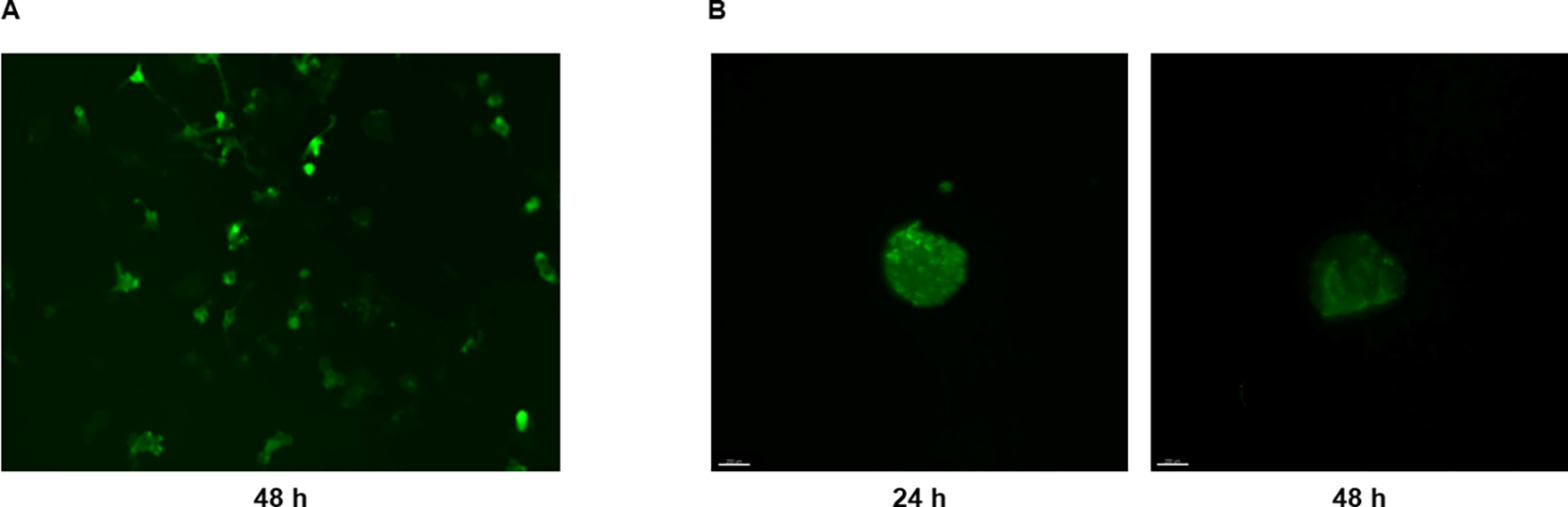
Fluorescent microscopy images of EGFP expression (green channel) in U87MG cells (A) and spheroids (B), after 24 and 48 h of the treatment of NPs loaded with 8% (w/w) of pDNA at 1 and 2.5 μg of pDNA, respectively (scale bar = 100 μm).

Furthermore, we conducted experiments to transfect U87MG glioblastoma cells with Pr/Dx NPs loaded with therapeutic miRNA-145, a microRNA known to be downregulated in various cancers, including glioma.^61^ As a control, cells were also transfected with a scrambled sequence. RT-PCR analysis revealed a 17-fold increase in miRNA-145 expression in U87MG cells compared with the scrambled control (Figure 8). These findings demonstrate that Pr/Dx NPs efficiently delivered exogenous miRNA-145 mimics into glioblastoma cells. Notably, the results surpass those reported for miRNA-145 delivery using chitosan–miRNA complexes,^62^ and other nanocomplexes, such as polyurethane-short-branch polyethylenimine (PU–PEI) both *in vitro* and *in vivo* using xenograft cancer models.^63^ While a direct comparison of efficacies with these references is not possible due to different experimental setups, these data provide a qualitative reference for the efficacy achieved. Overall, protamine NPs showed the effective delivery of nucleic acids. However, further studies will be necessary to evaluate the specific biological effects, such as cytotoxic response, that may result from miRNA-145 expression.

**Figure 8:**
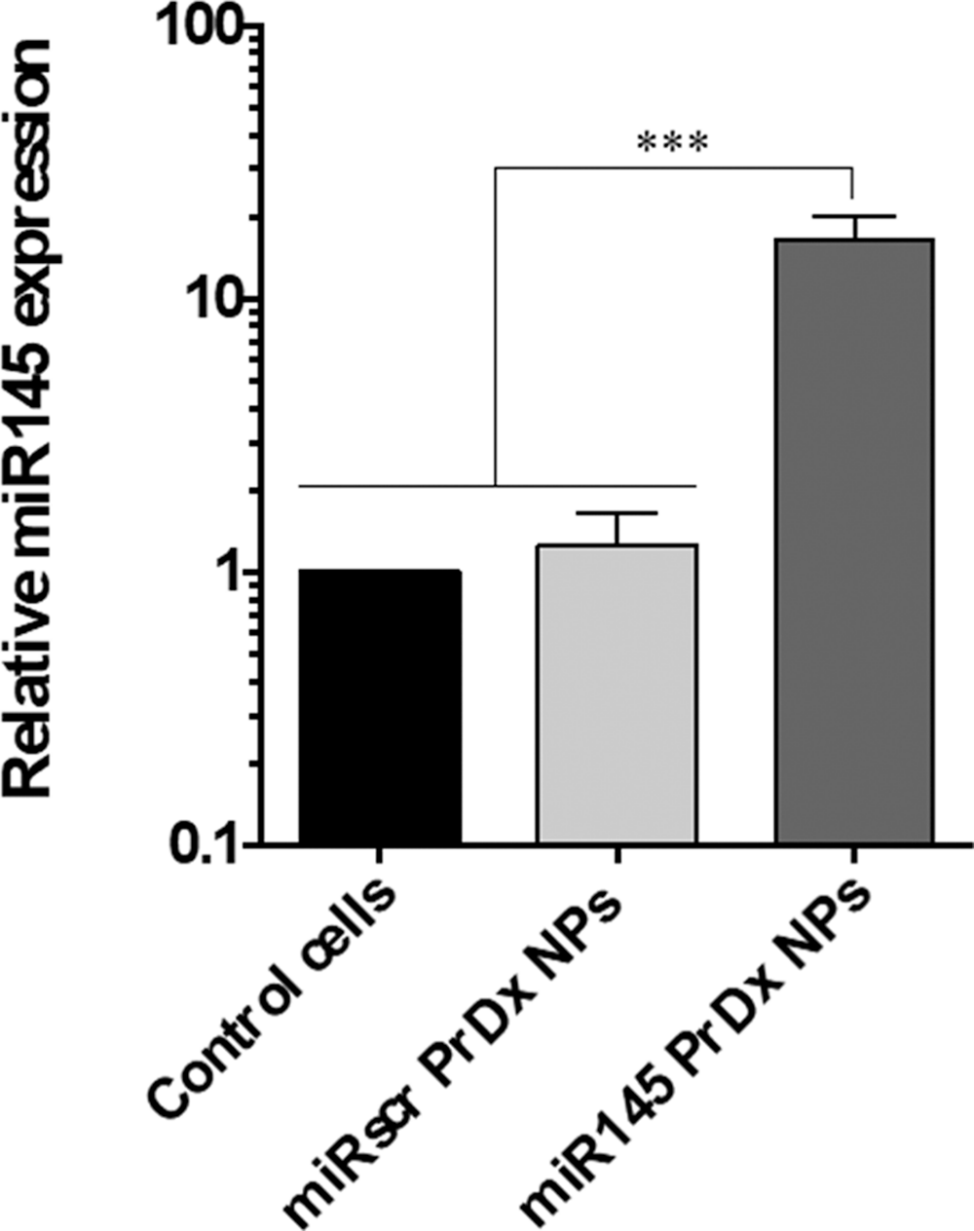
Relative expression levels of miRNA-145 in U87MG cells after transfection with miRNA-loaded Pr/Dx NPs at 5 μg or with a scrambled sequence. Data normalized against RNU6 (mean ± SD (*n* = 3)).

### 3.8 Evaluation of *In Vivo* Toxicity in Zebrafish Models

To complement our *in vitro* findings, the toxicity of the Pr/Dx NPs *in vivo* using the zebrafish model was also evaluated. Zebrafish (D. rerio) is becoming increasingly popular as an *in vivo* model for the toxicological and pharmacological screening of new compounds and nanomaterials. Within the field of nanotechnology, the zebrafish is a potential model for the evaluation of NPs due to its reduced cost, ease of husbandry, and high fecundity rates.^36,64^ The Fish Embryo Test (FET) for assessing acute and developmental toxicity has been standardized by the Organization for Economic Cooperation and Development (OECD) and has been shown to have a high correlation with the results in humans.

Overall, the results presented in Figure 9 and survival curves of Figure S8 indicated that the exposure of Pr/Dx NPs did not affect the survival of the zebrafish embryos, even at the highest concentrations and 96 h of incubation time. Thus, LC_50_ values for the formulation were above the highest concentrations tested. In contrast, mortality in the presence of 3,4-dichloroaniline was 100% at all concentrations studied (data not shown). These results were expected based on the previous *in vitro* cytotoxicity results obtained in the present work and previous studies carried out by Teijeiro-Valiño et al.^60^ where they also evaluated the toxicity profile of different protamine-based nanosystems in combination with other polymers such as hyaluronic acid. In these studies, they also observed a reduction in the percentage of mortality of zebrafish when the nanosystems were covered by a polymeric shell of protamine, thus verifying the good safety profile of this polymer. While the initial objective of this study was to determine whether the vehicle, in its nucleic acid-free form, exhibited inherent toxicity in advanced preclinical models such as zebrafish, we acknowledge the importance of future studies evaluating the toxicity of Pr/Dx NPs loaded with selected nucleic acids sequences. In general terms, the incorporation of nucleic acids into polymeric nanosystems has been shown to reduce their toxicity due to a decrease in positive charge, but other toxicity effects could possibly appear due to the expression/inhibition effect of the nucleic acid.

**Figure 9:**
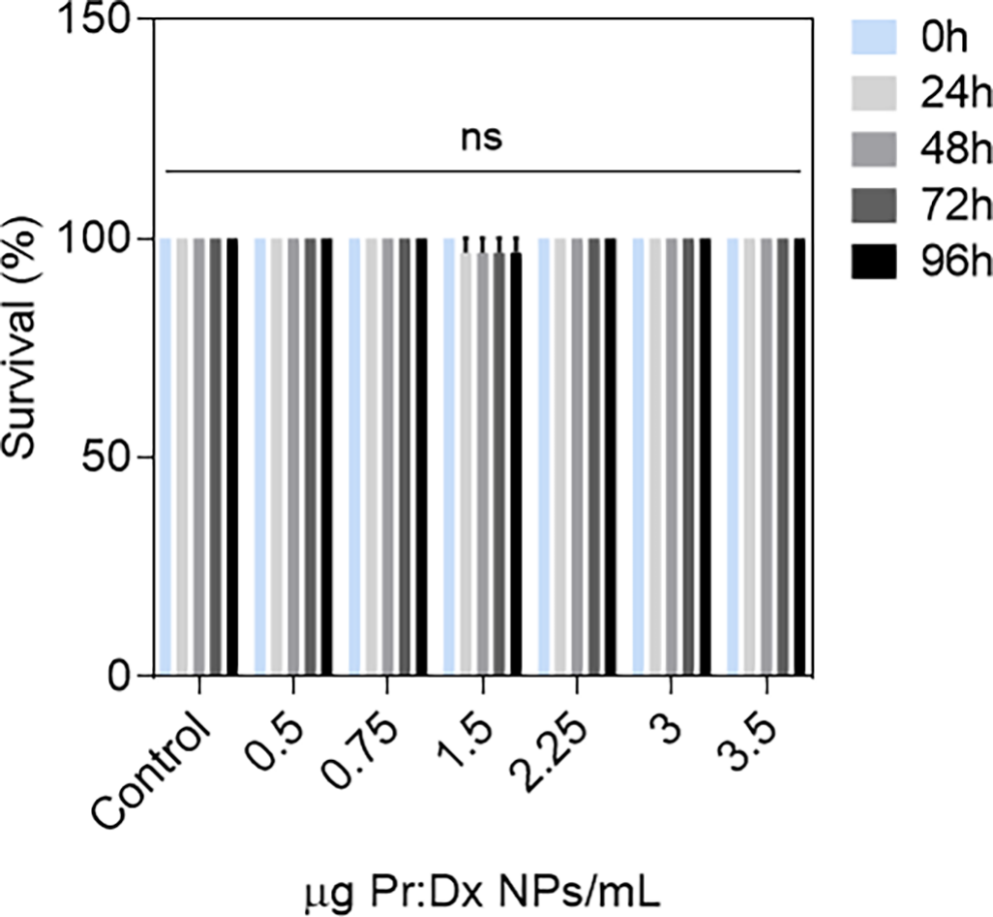
Percentage of zebrafish survival produced by blank 4:1 (w/w) Pr/Dx NPs at increasing concentrations after different times: 24, 48, 72, and 96 h of incubation (mean ± SEM (*n* = 3)).

## 4 Conclusions

The combination of protamine with polyanions such as dextran sulfate yields matrix-structured NPs that are useful as gene delivery nanosystems. This finding is proven by their highly flexible and tunable physicochemical characteristics that allow an effective association of different nucleic acids and satisfactory short- and long-term stability in different media and conditions. Furthermore, the absence of toxicity and the efficient internalization tested in advanced preclinical models of glioblastoma and in zebrafish highlight the potential of this formulation for gene delivery in glioblastoma. Additionally, Pr/Dx NPs have a high capacity to deliver a variety of gene therapies in glioblastoma cancer models, as demonstrated with miRNA-145. Further studies are needed for the optimization as a next step toward their preclinical development.

## Supplementary Material



## Notes

Complementary information about the patient-derived glioblastoma cell lines; stability of aqueous suspension of protamine NPs and in supplemented and non-supplemented cell culture medium; phase-contrast microscopy images of the morphology of glioblastoma spheroids before and after the treatment with protamine NPs; membrane-integrity assay of glioblastoma spheroids after the treatment with protamine NPs and its quantification by measuring the fluorescence signal of 7-AAD reagent; flow cytometry histograms of the total events of control and glioblastoma cells and spheroids; flow cytometry histograms to quantify the uptake of TAMRA-NPs in live events; flow cytometry images of positive TAMRA events of control, glioblastoma cells, and spheroids and their corresponding internalization histogram; table of the number of total positive events of control and glioblastoma cells treated with protamine NPs; and table of the number of total positive events of control and glioblastoma spheroids treated with protamine NPs (PDF)

## Abbreviations

ATCC: American Type Culture Collection
BBB: blood–brain barrier
BCA: bicinchoninic acid
CLSM: confocal scanning laser microscopy
CPP: cell-penetrating peptide
DAPI: 4′,6-diamino-2-phenylindole
DCR: derived count rate
DEPC: diethyl pyrocarbonate
DMEM: Dulbecco’s modified Eagle’s medium
DMSO: dimethyl sulfoxide
DPBS: Dulbecco’s phosphate buffered salt solution
Dx: dextran
EGFP: enhanced green fluorescence protein
EP: European Pharmacopeia
FBS: fetal bovine serum
FDA: Food and Drug Administration
FET: fish embryo acute toxicity test
GCE: glioma contrast-enhanced core cells
GIN: glioma invasive margin cells
HBSS: Hanks balanced salt solution
HEPES: *N*-2-hydroxyethylpiperazine-*N*-2-ethanesulfonic acid
HPLC: high-performance liquid chromatography
LC_50_: lethal concentration 50
LDA: laser Doppler anemometry
LMWP: low molecular weight protamine
LSFM: light sheet fluorescence microscopy
Luc: luciferase protein
Mw: molecular weight
NPs: nanoparticles
OECD: Organization for Economic Cooperation and Development
Opti-MEM: opti-minimum essential medium
P/S: penicillin–streptomycin
PBS: phosphate buffered salt solution
PCS: photon correlation spectroscopy
PDI: polydispersity index
PDMAEMA: poly­(2-*N*-(dimethylaminoethyl)­methacrylate)
PLL: poly­(l-lysine)
PEI: polyethylenimine
Pr: protamine
SDS: sodium dodecyl sulfate
SEM: scanning electron microscopy
STEM: scanning transmission electron microscopy
TAE: tris–acetate–EDTA
UV–vis: ultraviolet–visible
WHO: World Health Organization
WT: wild type
2D: two-dimensional
3D: three-dimensional
5-TAMRA: 5-carboxytetramethylrhodamine
7-AAD: 7-aminoactinomycin D
