# Supplementary material for: Protamine-Based Nanotherapeutics for Gene Delivery to Glioblastoma Cells
Source: Mol Pharm. 2025 Apr 2;22(5):2466–81. doi: 10.1021/acs.molpharmaceut.4c01269 (PMC12124719; doi:10.1021/acs.molpharmaceut.4c01269)
Supplement: Supplementary file 1 [file mp4c01269_si_001.pdf]

Supporting Information

Protamine-based nanotherapeutics for gene delivery to glioblastoma cells

Sheila Barrios-Esteban[a], Sonia Reimóndez-Troitiño[a], Pablo Cabezas-Sainz[c], María de la Fuente[d], Laura Sánchez[c], Ruman Rahman[e], Cameron Alexander[f], Marcos Garcia-Fuentes[a,b] and Noemi S. Csaba[a,b].*

[a]Center for Research in Molecular Medicine and Chronic Diseases (CiMUS), University of Santiago de Compostela, Campus Vida, 15706, Santiago de Compostela, Spain.

[b] Dept. Pharmacology, Pharmacy and Pharmaceutical Technology, School of Pharmacy, University of Santiago de Compostela, Campus Vida, 15706, Santiago de Compostela, Spain.

[c]School of Veterinary, University of Santiago de Compostela, Campus de Lugo, 27002, Lugo, Spain.

[d]Health Research Institute of Santiago de Compostela (IDIS),15706, Santiago de Compostela, Spain.

[e]Children's Brain Tumor Research Centre (CBTR) and Biodiscovery Institute (BDI), University of Nottingham, University Park, Nottingham, NG7 2RD, United Kingdom.

[f]School of Pharmacy, [b]Boots Science Building (BSB), University of Nottingham, East Dr, Nottingham, NG7 2TQ, United Kingdom.

[*]Email: noemi.csaba@usc.es

Table S1. Complementary information of the three patient-derived glioblastoma cell lines: GIN-8, GIN-28, and GCE-28.

Cell line	Gender/year	Tumor type	Promoter	Treatment	Death after surgery
**GIN-8**	Female/54	Wild-type IDH (primary glioblastoma)	Intact ATRX 0% MGMT promoter methylation	90% resection plus Gliadel wafers. 60Gy radiotherapy. Concurrent/adjuvant temozolomide	5 months
**GIN-28**	Male/71			99% resection. No adjuvant therapy (patient choice)	3 months
**GCE-28**	Male/71			99% resection. No adjuvant therapy (patient choice)	3 months

A

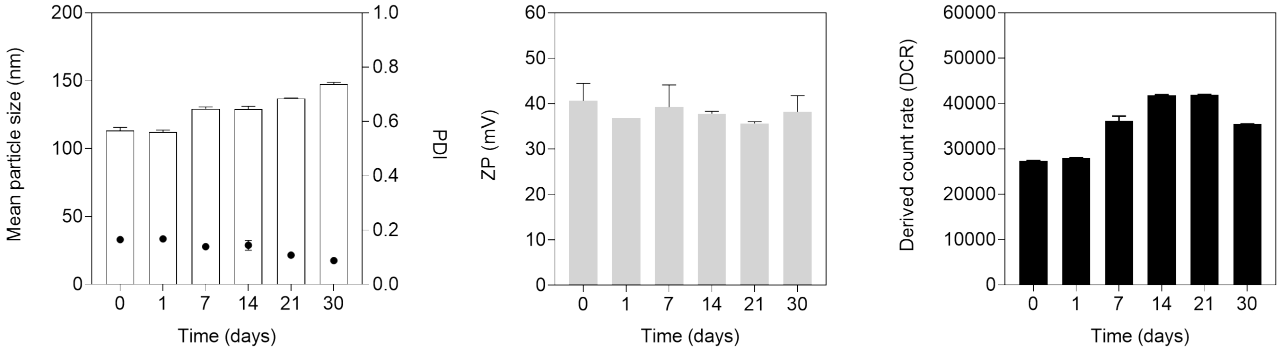

B

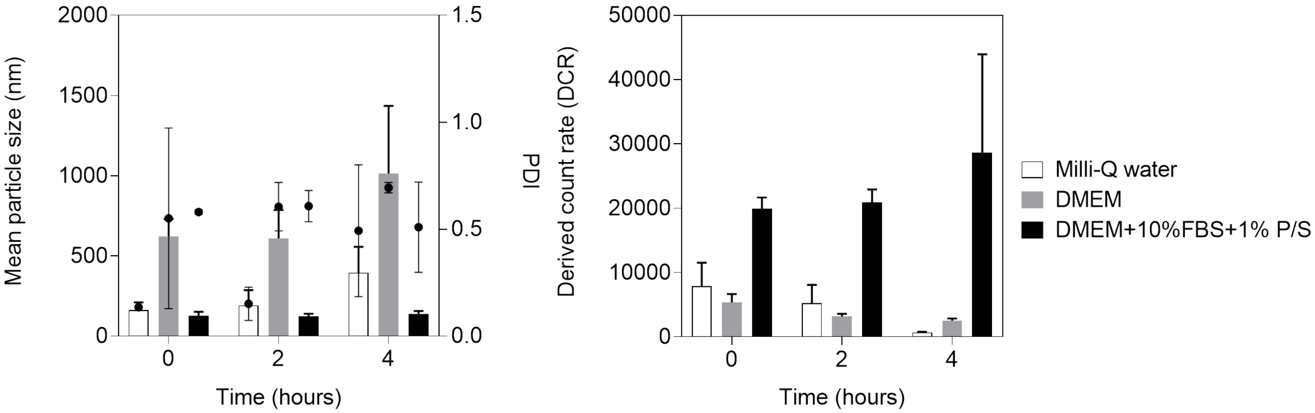

Figure S1. Stability of aqueous suspensions of blank 4:1 (*w/w*) Pr:Dx NPs at 4 °C for 30 days (**A**) and in supplemented and non-supplemented DMEM cell culture medium at 0, 2 and 4 h at 37 °C (**B**) measuring the size (bars) and polydispersity index (PDI) (dots), zeta potential and derived count rate (DCR) (Mean ± SD (n≥ 2)).

A

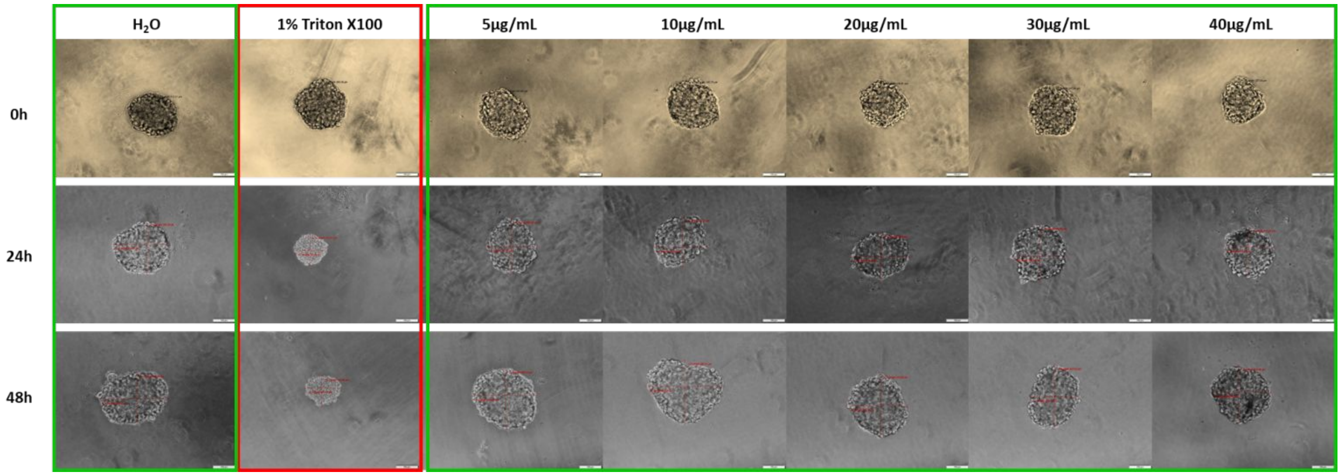
H2O
1% Triton X100
5μg/mL
10μg/mL
20μg/mL
30μg/mL
40μg/mL
0h
24h
48h

B

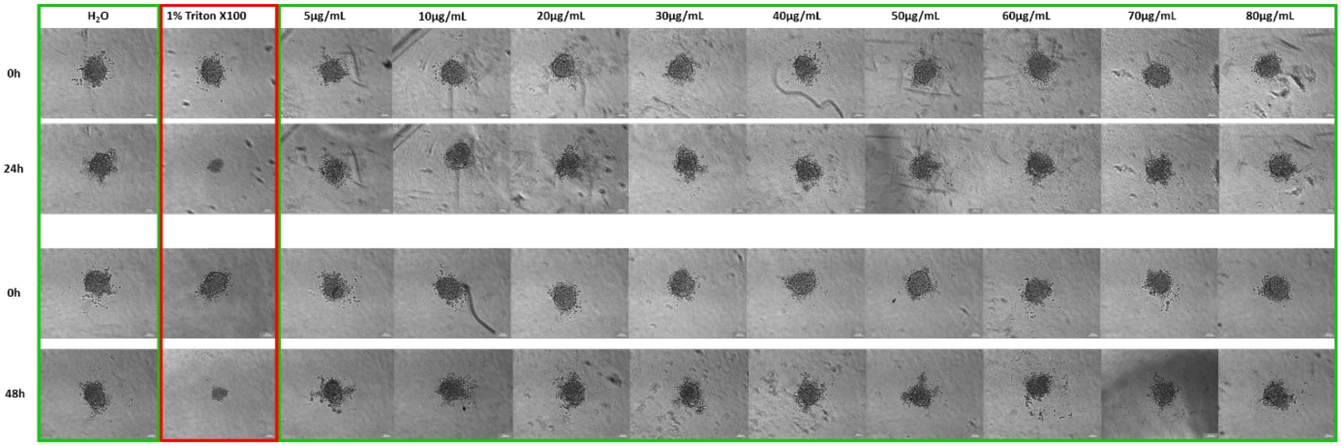
H2O
1% Triton X100
5μg/mL
10μg/mL
20μg/mL
30μg/mL
40μg/mL
50μg/mL
60μg/mL
70μg/mL
80μg/mL
0h
24h
0h
48h

C

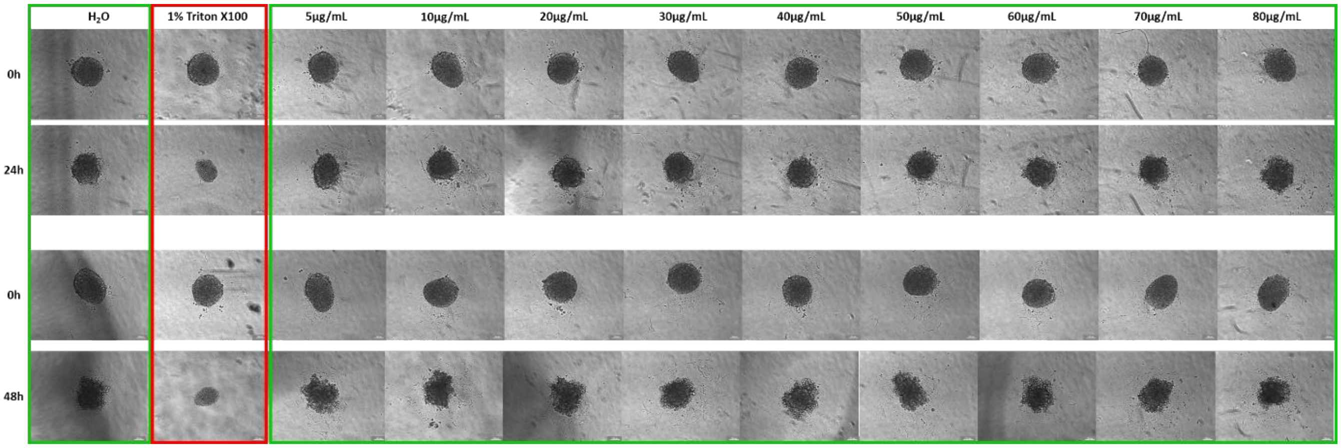
H$_2$O
1% Triton X100
5µg/mL
10µg/mL
20µg/mL
30µg/mL
40µg/mL
50µg/mL
60µg/mL
70µg/mL
80µg/mL
0h
24h
0h
48h

D

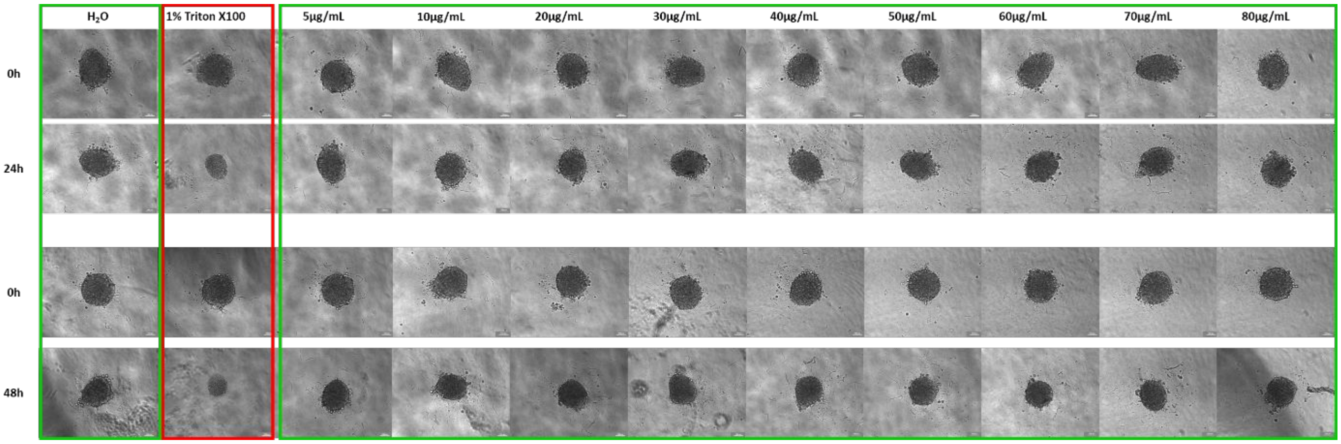
H$_2$O
1% Triton X100
5µg/mL
10µg/mL
20µg/mL
30µg/mL
40µg/mL
50µg/mL
60µg/mL
70µg/mL
80µg/mL
0h
24h
0h
48h

Figure S2. Phase contrast microscopy images of the morphology of U87MG (**A**), GIN-8 (**B**), GIN-28 (**C**) and GCE-28 (**D**) spheroids before and after 24 h and 48 h of treatment of increasing concentrations of blank 4:1 (*w/w*) Pr:Dx NPs, from 5 to 40 µg/mL, and from 5 to 80 µg/mL, respectively (magnification 10x, scale bar= 100 µm).

A

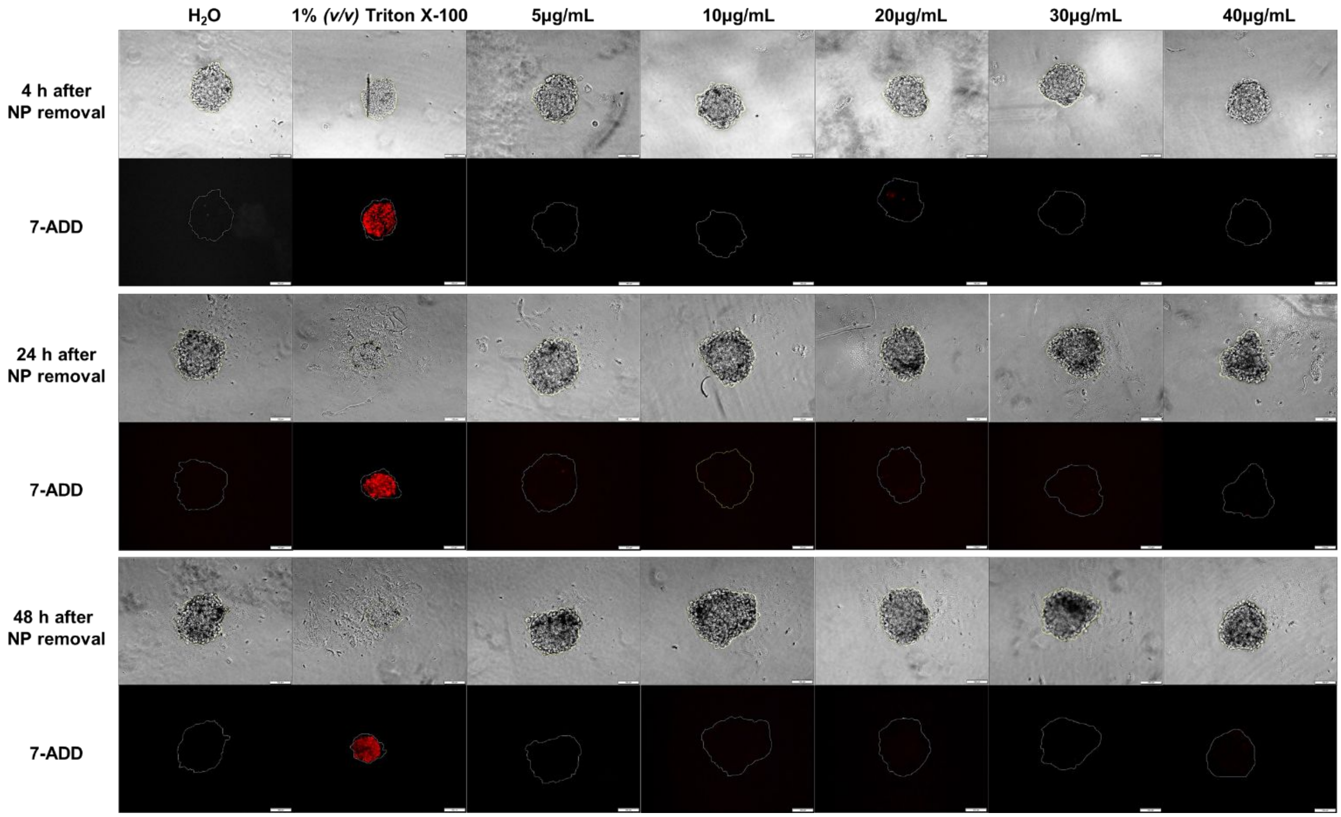

B

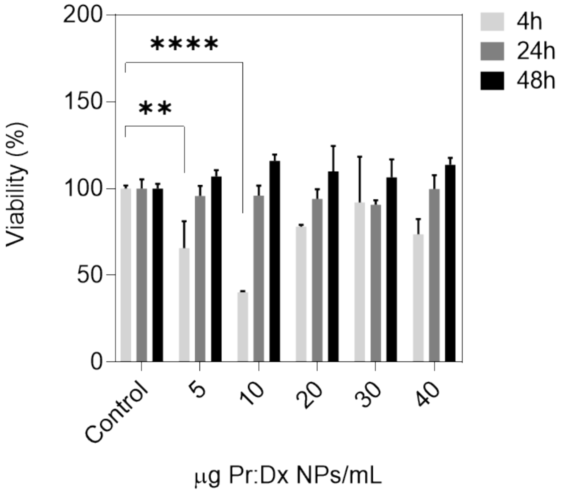

Figure S3. Membrane-integrity assay of U87MG spheroids after 24 h and 48 h of the removal of different concentrations of blank 4:1 (*w/w*) Pr:Dx NPs, analyzed by fluorescence microscopy (magnification 10x, scale bar= 100 μm) (**A**) and quantified by measuring the fluorescence signal of 7-AAD reagent (Mean ± SD (n= 3)) (**B**).

A

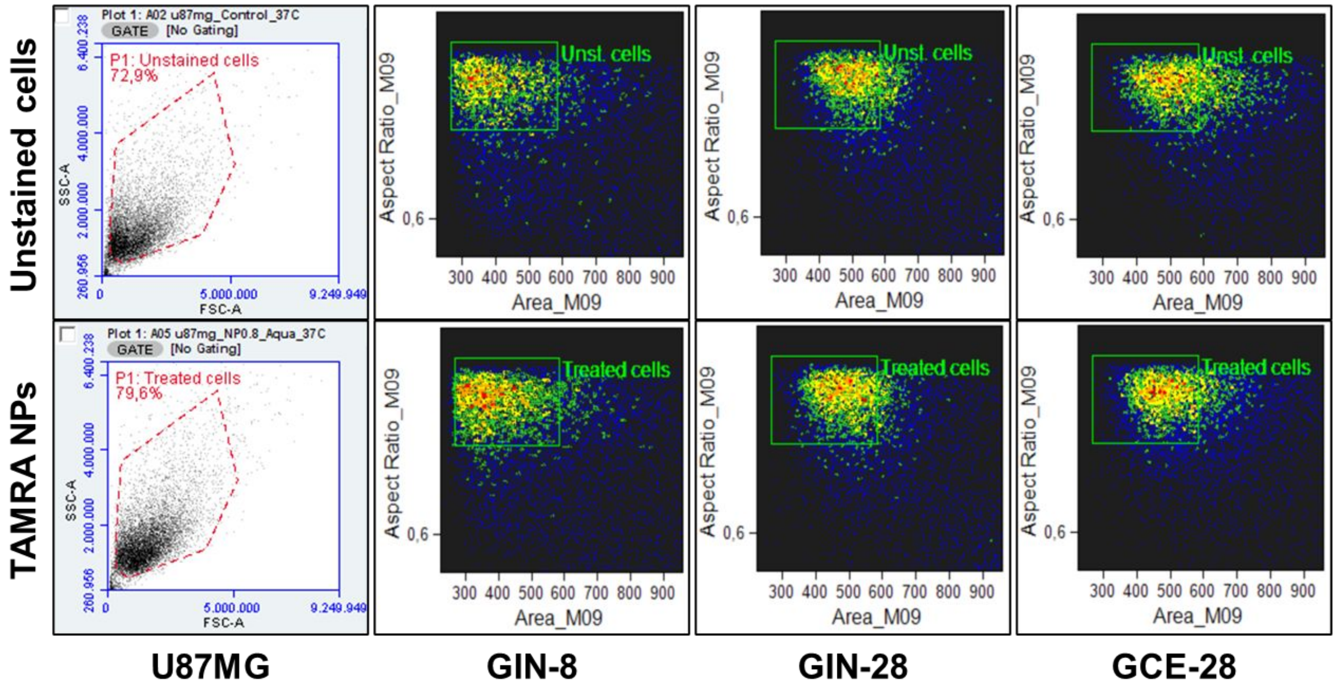

Unstained cells
TAMRA NPs
Plot 1: A02 u87mg_Control_37C
GATE [No Gating]
P1: Unstained cells
72,9%
Plot 1: A05 u87mg_NP0.8_Aqua_37C
GATE [No Gating]
P1: Treated cells
79,6%
SSC-A
FSC-A
6.400.238
4.000.000
2.000.000
260.956
0
5.000.000
9.249.949
Aspect Ratio_M09
Area_M09
0,6
300 400 500 600 700 800 900
Unst. cells
Treated cells
U87MG
GIN-8
GIN-28
GCE-28

B

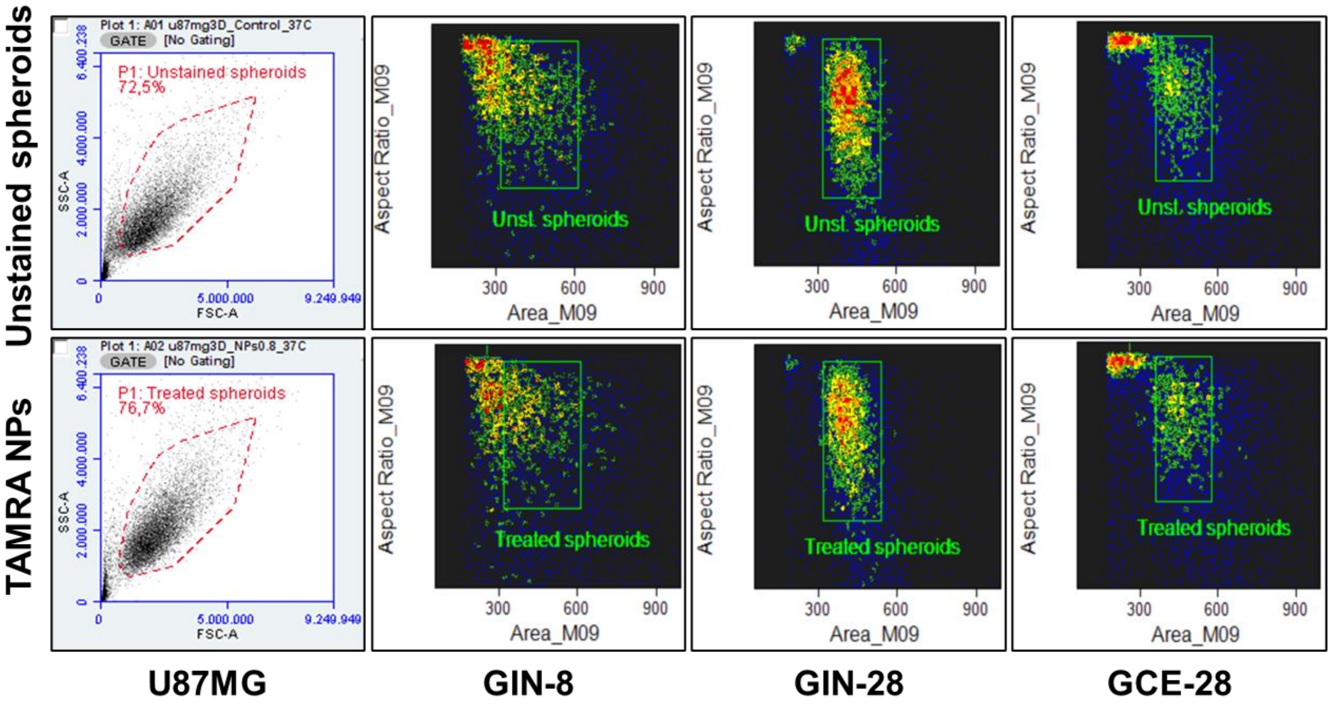

Figure S4. Flow cytometry histograms of the total events without debris of control and glioblastoma cells (**A**) and spheroids (**B**) treated with blank 4:1 (*w/w*) Pr:Dx NPs (7 µg/cm²) to analyze the NP-uptake.

A

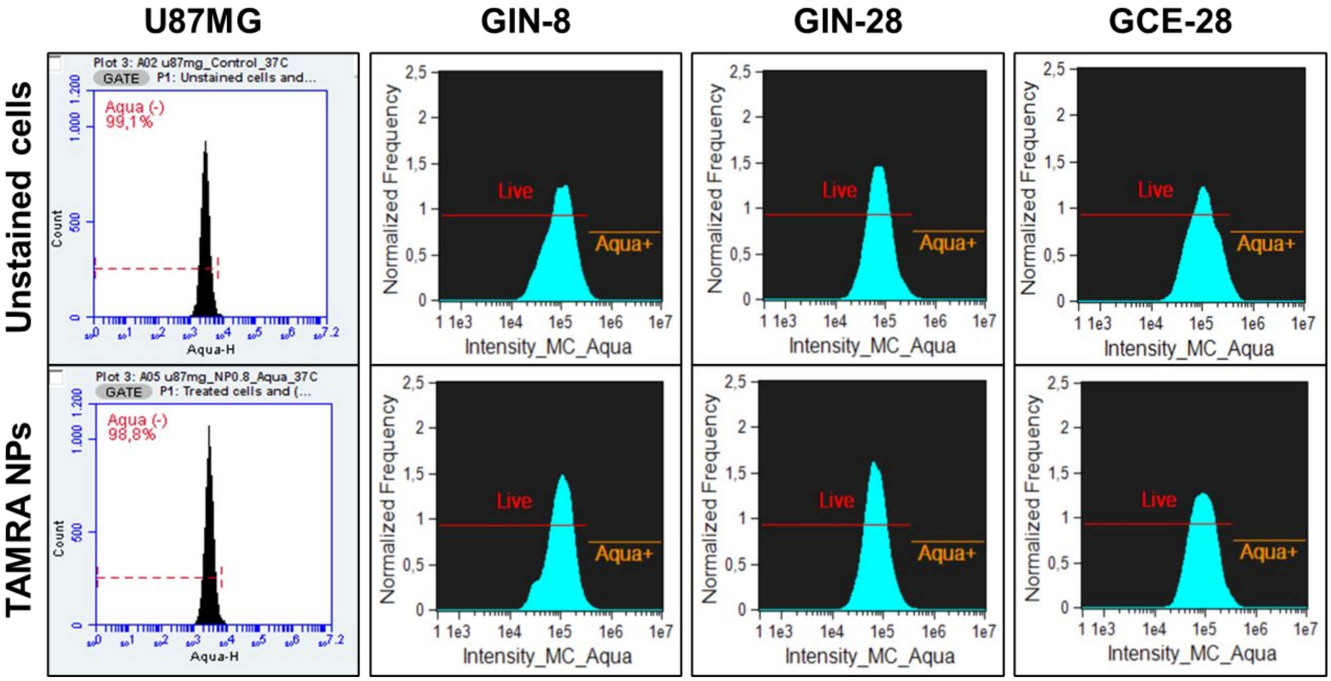
U87MG
GIN-8
GIN-28
GCE-28
Unstained cells
TAMRA NPs
Plot 3: A02 u87mg_Control_37C
GATE P1: Unstained cells and...
Aqua (-)
99,1%
Count
Aqua-H
Plot 3: A05 u87mg_NP0.8_Aqua_37C
GATE P1: Treated cells and (...
Aqua (-)
98,8%
Normalized Frequency
Live
Aqua+
Intensity_MC_Aqua

B

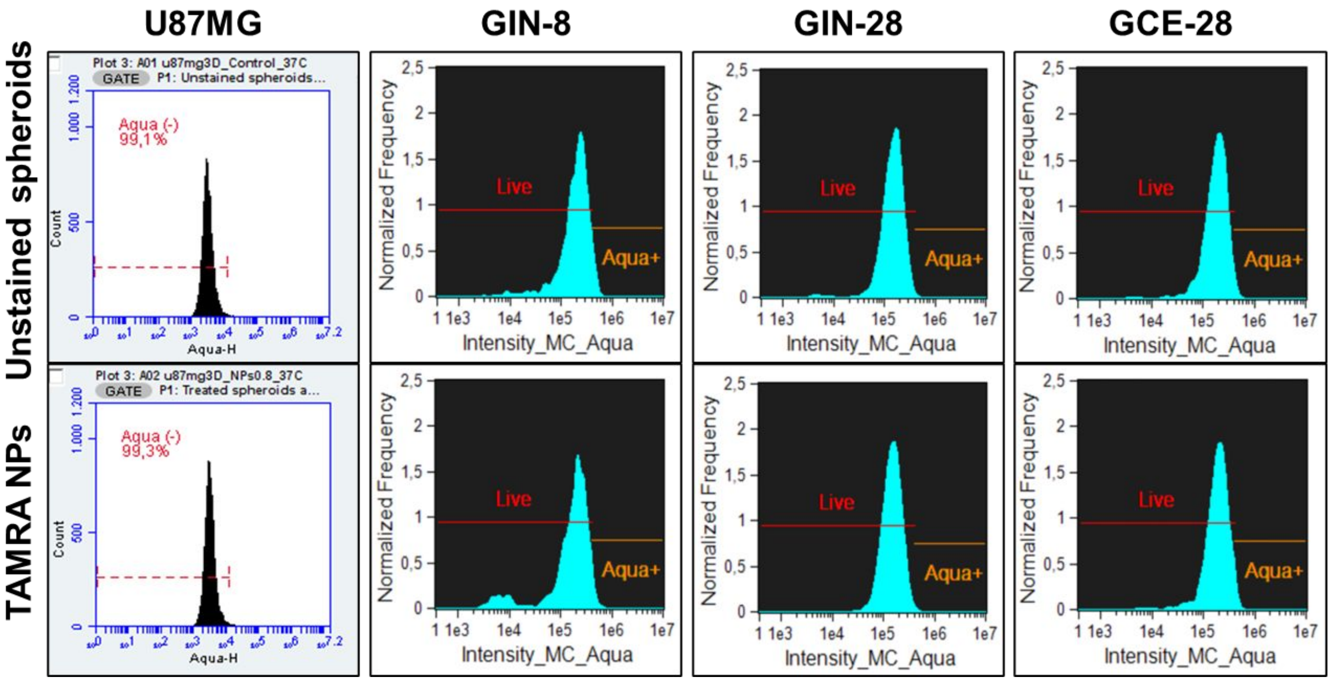

Figure S5. Flow cytometry histograms to quantify the uptake of fluorescently TAMRA-NPs (7 µg/cm^2) in living U87MG, GIN-8, GIN-28, GCE-28 cells (**A**), and spheroids (**B**) after 4 h post-treatment using LIVE/DEAD™ Fixable Aqua Dead Cell Stain as a viability reagent.

A

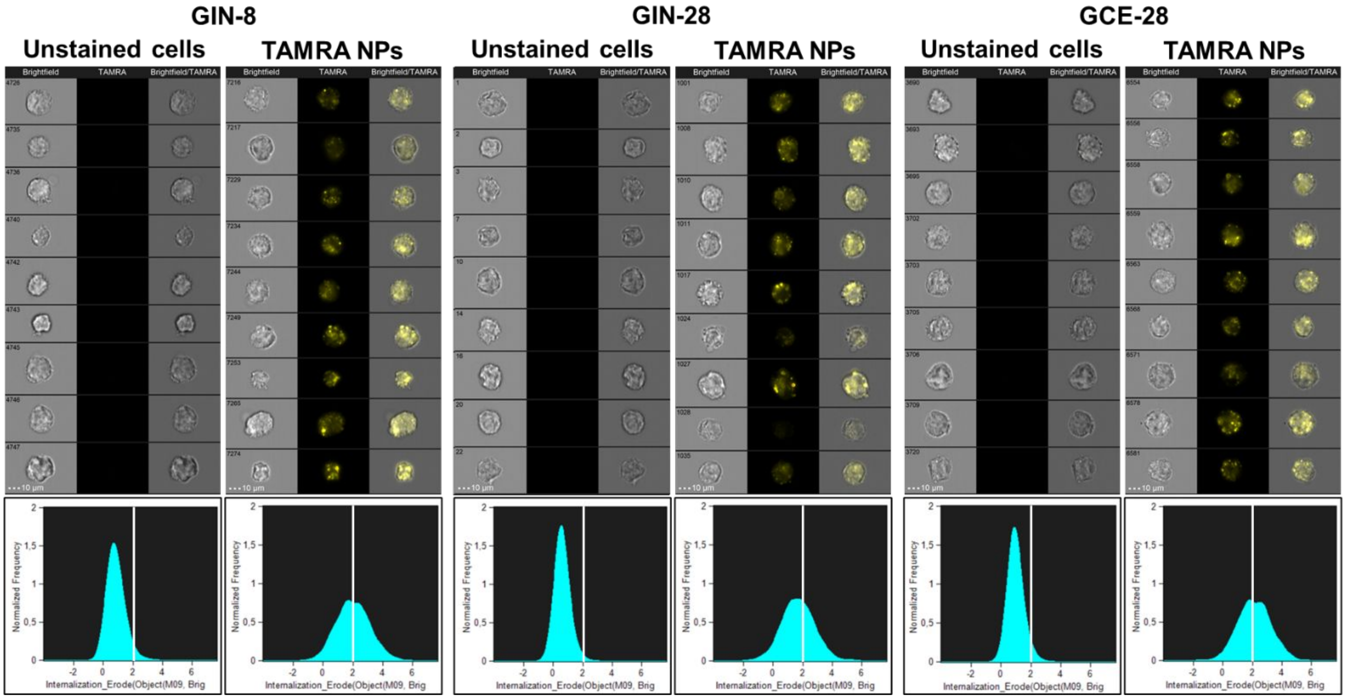
GIN-8
Unstained cells
TAMRA NPs
GIN-28
Unstained cells
TAMRA NPs
GCE-28
Unstained cells
TAMRA NPs
Brightfield
TAMRA
Brightfield/TAMRA
10 µm
Normalized Frequency
Internalization_Erode(Object(M09, Brig

B

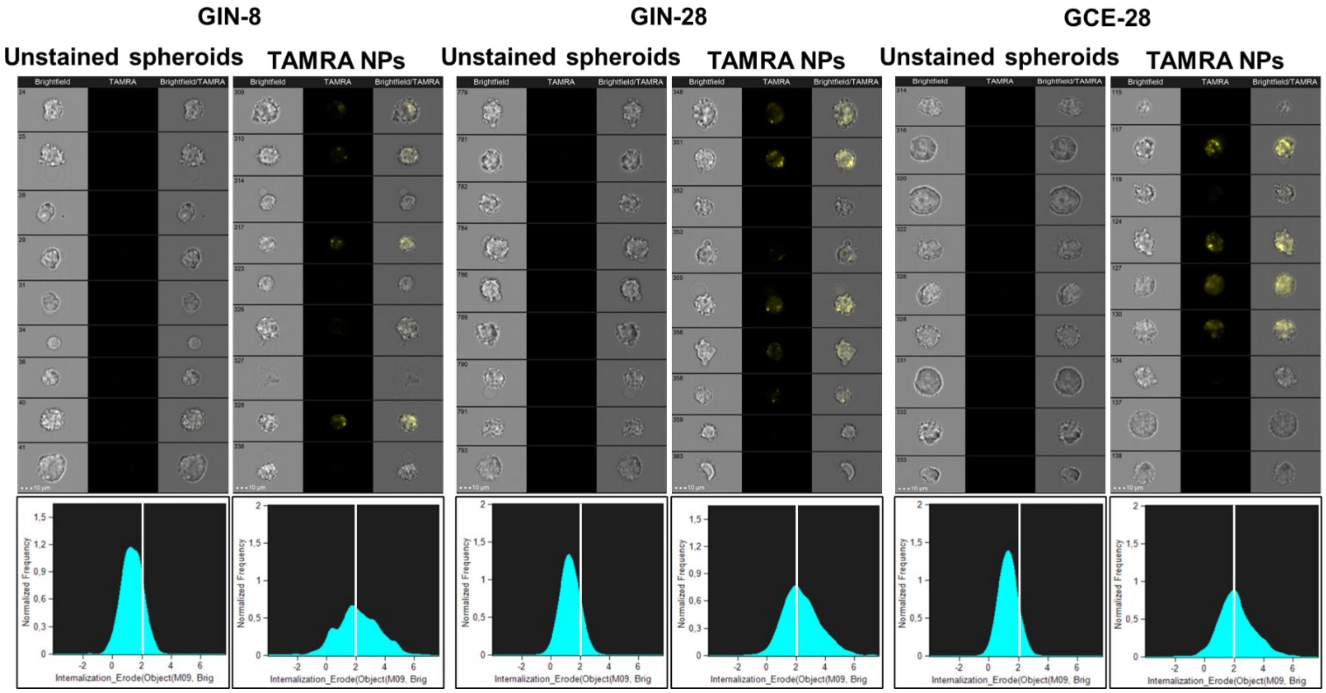

Figure S6. Flow cytometry images of positive 5-TAMRA events of control (grey) and primary patient-derived glioblastoma cells (**A**) and spheroids (**B**) treated with fluorescent protamine NPs (7 µg/cm^2, yellow channel) and their corresponding internalization histogram (values ≥ 2 indicated NP-internalization).

Table S2. Number of total positive events of control and glioblastoma cells treated with blank 4:1 (*w/w*) Pr-TAMRA:Dx NPs expressing by percentage and measuring their Mean Fluorescence Intensity (MFI).

Cell line	Total (+) 5-TAMRA events		% (+) 5-TAMRA events		MFI (+) 5-TAMRA events	
	control	sample	control	sample	control	sample
U87MG	73	7915	1	99.4	41,725	145,580
GIN-8	10	1205	0.31	99.8	12,489	334,308
GIN-28	1	3227	0.02	100	14,561	291,450

**GCE-28**	24	2297	0.66	99.7	16,142	305,253

Table S3. Number of total positive events of control and glioblastoma spheroids treated with blank 4:1 (*w/w*) Pr-TAMRA:Dx NPs expressing by percentage and measuring their Mean Fluorescence Intensity (MFI).

**Cell line**	**Total (+) 5-TAMRA events**		**% (+) 5-TAMRA events**		**MFI (+) 5-TAMRA events**	
	**control**	**sample**	**control**	**sample**	**control**	**sample**
**U87MG**	74	7,666	1	99.9	58,904	289,726
**GIN-8**	43	457	4.7	62.9	11,454	72,790
**GIN-28**	3	1,613	0.09	58.8	10,771	66,842
**GCE-28**	12	744	0.75	55.6	11,269	76,936
